# Prediction of regional functional impairment following experimental stroke via connectome analysis

**DOI:** 10.1038/srep46316

**Published:** 2017-04-13

**Authors:** O. Schmitt, S. Badurek, W. Liu, Y. Wang, G. Rabiller, A. Kanoke, P. Eipert, J. Liu

**Affiliations:** 1 Department of Anatomy, University of Rostock, Germany; 2 Department of Neurological Surgery, UCSF, San Francisco, CA 94158, USA; 3 Department of Neurological Surgery, SFVAMC, San Francisco, CA 94158, USA; 4 Department of Neurological Surgery, Beijing Tiantan Hospital, Capital Medical University, Beijing, 100050, PR China

## Abstract

Recent advances in functional connectivity suggest that shared neuronal activation patterns define brain networks linking anatomically separate brain regions. We sought to investigate how cortical stroke disrupts multiple brain regions in processing spatial information. We conducted a connectome investigation at the mesoscale-level using the neuroVIISAS-framework, enabling the analysis of directed and weighted connectivity in bilateral hemispheres of cortical and subcortical brain regions. We found that spatial-exploration induced brain activation mapped by Fos, a proxy of neuronal activity, was differentially affected by stroke in a region-specific manner. The extent of hypoactivation following spatial exploration is inversely correlated with the spatial distance between the region of interest and region damaged by stroke, in particular within the parietal association and the primary somatosensory cortex, suggesting that the closer a region is to a stroke lesion, the more it would be affected during functional activation. Connectome modelling with 43 network parameters failed to reliably predict regions of hypoactivation in stroke rats exploring a novel environment, despite a modest correlation found for the centrality and hubness parameters in the home-caged animals. Further investigation in the inhibitory versus excitatory neuronal networks and microcircuit connectivity is warranted to improve the accuracy of predictability in post-stroke functional impairment.

Post-stroke cognitive impairment in the domain of short- and long-term memory is not uncommon, yet the mechanism underlying this memory deficit is unclear. The hippocampus is involved in memory function, but direct ischemic lesions strategic to the hippocampus or parahippocampal regions are rarely observed in middle cerebral artery (MCA) stroke^1^,^2^,^3^. Previously, we found that stroke by distal occlusion of MCA (dMCAO) produced injury restricted to the cortex and mild memory impairment^4^. A number of cortical areas affected by dMCAO including the parietal cortex are strongly interconnected by other cortical, subcortical, diencephalic, mesencephalic and brainstem regions of the ipsilateral side of the occlusion as well as the contralateral hemisphere. It is well known that the interplay of several brain regions is crucial for learning and memory. Recent advances in functional connectivity also suggest that shared neuronal activation patterns define brain networks linking anatomically separate brain regions. Remote brain areas that are not directly affected by ischemia can still undergo functional changes caused by oedema, spreading depression of neuronal activity, changes in projection pathways, or by reactive plasticity.

Previous studies showed that several limbic brain regions were activated when normal rats were exposed to a novel environment, and brain lesions produced hypoactivity within connected brain regions that were associated with spatial memory^5^,^6^,^7^. Understanding the relationship between infarct regions and changes of regional neuronal activity may provide insight in the flow of information or processing in the brain, as well as functional reorganization or compensation of cortical and subcortical regions^8^,^9^. The knowledge in lesion location and network structure may also enable the identification of brain regions undergoing functional changes and the nature of functional impairment, making it possible to develop targeted intervention.

The present study sought to investigate how ischemic injury in the parietal cortex disrupts multiple brain regions in processing spatial information. First, neuronal activation induced by spatial exploration was mapped in rats that underwent stroke or sham surgery with the immediate early gene Fos, a proxy of neuronal activity, the expression of which is induced under conditions of learning and memory^5^,^10^. The relationship between damaged regions and changes of regional neuronal activation depicted by Fos expression was analysed in neuroVIISAS^11^,^12^, a rat connectome framework at the mesoscale level based on tract-tracing bilateral connectivity with semiquantitative weights. We then examined whether regions with changes in neuronal activity during spatial task could be predicted via the connectome analysis and modelling. Lastly we discerned the relationship between the extent of neuronal hypoactivation following spatial exploration and the spatial distance between the region of interest and the region damaged by stroke. We found that there was a region-specific reduction in brain activation in several hippocampal, parahippocampal and thalamic regions during spatial exploration, and the extent of neuronal hypoactivation of a particular region of interest was inversely correlated with its spatial distance to the infarct regions in the parietal cortex.

## Material and Methods

This study was conducted in accordance with the animal care guidelines issued by the National Institutes of Health and approved by the San Francisco Veterans Affair Medical Center Animal Care and Use Committee. The identity of each animal with respect to treatment was concealed to experimenters who conducted the procedures and analysis.

### Experimental stroke

Stroke was induced unilaterally in male Sprague-Dawley rats (N = 21, 2.5 month of age, Charles River, CA) under isoflurane/O_2_/N_2_O (1.5/30/68.5%) according to the well-established distal middle cerebral artery occlusion (dMCAO) method^13^. Briefly, the main trunk of the left MCA was ligated just underneath the rhinal fissure with a 10-0 suture, and the bilateral common carotid arteries (CCA) were occluded for 60 minutes with 4-0 sutures. The sutures were then removed to restore blood flow, and the cervical incision was closed. Sham-operated rats (N = 19) did not receive occlusion of either the MCA or the CCAs.

### Spatial exploration and assessing neural activation

Rats were either maintained in standard housing conditions (“home”) (N = 22) or underwent a spatial exploration task (“expl”) (N = 18), consisting of 15-minute exploration of an open field on a circular table (120 cm in diameter) with a large ball placed on the table each on days 4 and 5 after MCAO^5^. The spatial configuration of the environment was altered between sessions post MCAO simply by changing the position of the large ball from the center (days 4) to the periphery (days 5) of the table with everything else remained the same. Ninety minutes after the exploration task (or at the same time of the day for “home cage” controls), rats were perfused with 4% paraformaldehyde and 40 μm coronal sections were stained for the expression of Fos (rabbit-anti-Fos, Oncogene Science, 1:5000)^14^. Fos-positive cells were counted manually under a light microscope at 40x magnification using the NIH Image J cell counting plugin tool. Three sequential serial sections (480 μm apart) were analysed per animal and the counts were averaged and expressed as number of cells/mm^2^.

### Analysis of Fos expression

To calculate Fos differences between treatment groups and hemispherical locations, we defined 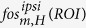 as the mean Fos expression in the region of interest (ROI) ipsilateral (*ipsi*) to dMCAO, among rats with ischemic stroke (*m*) and remained in the home cage (*H*), while 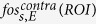 as the mean Fos expression in the region of interest contralateral to dMCAO, among rats with sham surgery (*s*) and subjected to spatial exploration (*E*).

#### Stroke effect


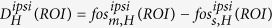
 represents the difference of the Fos expressions in the ROI ipsilateral to dMCAO between stroke rats and sham-operated rats remained in the home cage environment (*H*). Analogue is the definition for the rats in the exploration environment with the lower index *E*.

#### Exploration effect


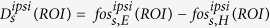
 compares the difference of the Fos expressions between sham rats remained in the home cages and those subjected to exploration in the ipsilateral region (in this case, no different from the contralateral side). Analogue is the definition for the rats in the dMCAO group with the lower index *m*.

The relative differences *Dr* of the ipsilateral side are defined as follows:


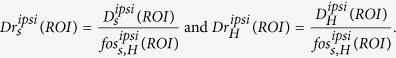


To integrate the variability of the Fos expressions within the groups a second relative difference is defined, where the difference is divided by the standard deviation (σ).
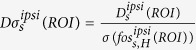
.

An absolute value below one means that the difference is below the SEM. The mean of the absolute values of the differences of all *n* regions is:


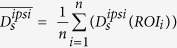


### Analysis of connectivity

The network-analysis was performed by using the neuroVIISAS framework^11^. The rat nervous system connectome project within neuroVIISAS was applied to compute local network parameters^12^ of regions that show Fos changes. This connectome was built by collating connections from publications that describe results of tract tracing experiments in normal adult rat. Such a meta-analysis is an established method^15^,^16^,^17^ to generate bilateral, directed and weighted mesocale-connectomes. In this particular investigation 4834 publications were evaluated, describing more than 440000 central and peripheral connections. Three dimensional visualization was performed within neuroVIISAS using a stereotaxic atlas of the rat^18^. The colour code for each Fos-expressing region is defined based on the hierarchy in neuroontology defined in the neuroVIISAS, and is kept consistent throughout all the figures.

A number of local network parameters are modestly correlated with Fos expression and defined below, such as the eigenvector centrality (EC), subgraph centrality (SC), hubness and authoritativeness. If the correlation coefficient of a local network parameter with Fos expression changes is sufficiently large, it is thus trustworthy to use the neuroVIISAS connectome model to predict Fos changes as an index of functional activation (or impairment) following the removal of lesioned regions in the network. In other words, after removing the damaged region(s), the values of the specific local network parameters can be calculated for the remaining regions. Should a large correlation or coherence between a network parameter and Fos expression be found, the functional activation of a given region of interest can be predicted with a certain probability. The centrality of a region, also known as a node, is its relative importance within a network in terms of its connections. The eigenvector centrality (Gould centrality, Bonacich’s centrality) is a measure of influence of a node in a network^19^. Important nodes with large eigenvector centralities are connected to important neighbours. The subgraph centrality was introduced by Estrada^20^ and is based on the participation of each node in all subgraphs of a network, whereby smaller subgraphs are given more weight than larger ones. The definition of hub and authority is circular. A hub is a node that points to many authorities and an authority is a node that has numerous input connections from many hubs^21^. Hubness was computed according to Kleinberg^21^ with modifications. In order to identify hubs and authorities the iterative method of Kleinberg was used to break the circularity. Nonnegative authority weights and hub weights were computed and normalized to their squares sum. Those with larger authority or hub weights are more likely to be hubs and authorities, respectively. The mutually reinforcing relationship between hubs and authorities has been numerically determined by increasing authority weight if the region p is connected to many regions with large authority weights then it should receive a large hub weight. If the region p is connected to many regions with large hub weights then it should receive a large authority weight, although neither hub nor authority weights is related connection weights. The vulnerability of a given region also reflects the importance of the region with regard to its connections to other connected regions, in which by removing or damaging this given region would enlarge the mean graph theoretical distance among the remaining regions. The vulnerability calculation can be compared with an artificial removal of a region from a connectome. A quantitative change of a network following the removal of a region can be estimated by calculating the average closeness before and after the removal. If the vulnerability value is positive, then the average closeness decreases. Otherwise a negative vulnerability indicates an increase of the average closeness (e.g. removal of a region node that has inputs or outputs only).

Abbreviations of local network parameters are as following (formal definition and interpretations are given in ref. 22): REC: Number of reciprocal connections, AvgRang: Average rank, DGAll: Number of inputs and outputs of a node, DGOut: Number of outputs of a node, DGIn: Number of inputs of a node, CDC: Convergence-divergence coefficient, LatAll: Laterality coefficient of inputs and outputs, LatOut: Laterality coefficient of outputs, LatIn: Laterality coefficient of inputs, LatRec: Laterality coefficient of reciprocal connections, Katz: Katz status index, LCircle: Shortest path to a node, Triag: Number of triangles around a node, CyclC: Cyclic coefficient, EccOut: Eccentricity for output. EccIn: Eccentricity for input, CluCOut: Cluster coefficient for output, CluCIn: Cluster coefficient for input, CluCAll: Cluster coefficient for input and output, CluCTriag: Cluster coefficient triangle-based, CluC2: Hierarchical cluster coefficient of level 2, AvgDGnb: Average degree of all neighbors of a node, VCDG:Variation coefficient of neighbor degree, Lev: Levarage, Loc Locality, CCOut: Closeness centrality for output, CCIn: Closeness centrality for input, BC: Betweeness centrality, IN: Number of shortest paths of length 2, EC: Eigenvector centrality, SC: Subgraph centrality, PRC: Pagerank centrality. FC: Flow coefficient, Stress: Stress, Shapley: Shapely index, ZOut: Z-score for output, ZIn: Z-score for input, ZAll: Z-score for input and output, PCOut: Participation coefficient output, PCIn: Participation coefficient input, PCAll: Participation coefficient input and output, RadOut: Radiality output, RadIn: Radiality input, CenOut: Centroid output value, CenIn: Centroid input value, Hub: Hubness, Aut: Authority, Knot: Knotty center.

### Ranking of network parameters

Ordinal ranking has been applied to local network parameters of damaged and fos regions. For each region and each local network parameter a distinct ordinal number has been attributed. For example, all regions obtained a rank number after sorting their degree all parameter. The regions with the largest degree all parameter obtained the lowest rank number (“row numbering”). This was repeated for all local parameters and an average rank was determined for each region. Since some graph theoretic measures are strongly inversely correlated, local parameters like the Shapley-index in which a more negative value correlates with a larger connectional importance of a region, thus have been ranked high by taking this inverse correlation relationship into account. Vice versa, the largest positive values of the cluster coefficient also correspond to top ranks with lowest rank numbers.

### Statistical Analysis

Data are expressed as mean ± SEM and examined statistically for differences between groups by analyses of variance (ANOVAs) followed by *post hoc* paired comparisons with Bonferroni adjustment when appropriate (SAS 5.0.1, SAS Institute Inc., Cary, NC). Values of p < 0.05 were considered as significant. Sample size was 8-11/group.

## Results

### Spatial exploration increases Fos expression in multiple brain regions

**Figure 1:**
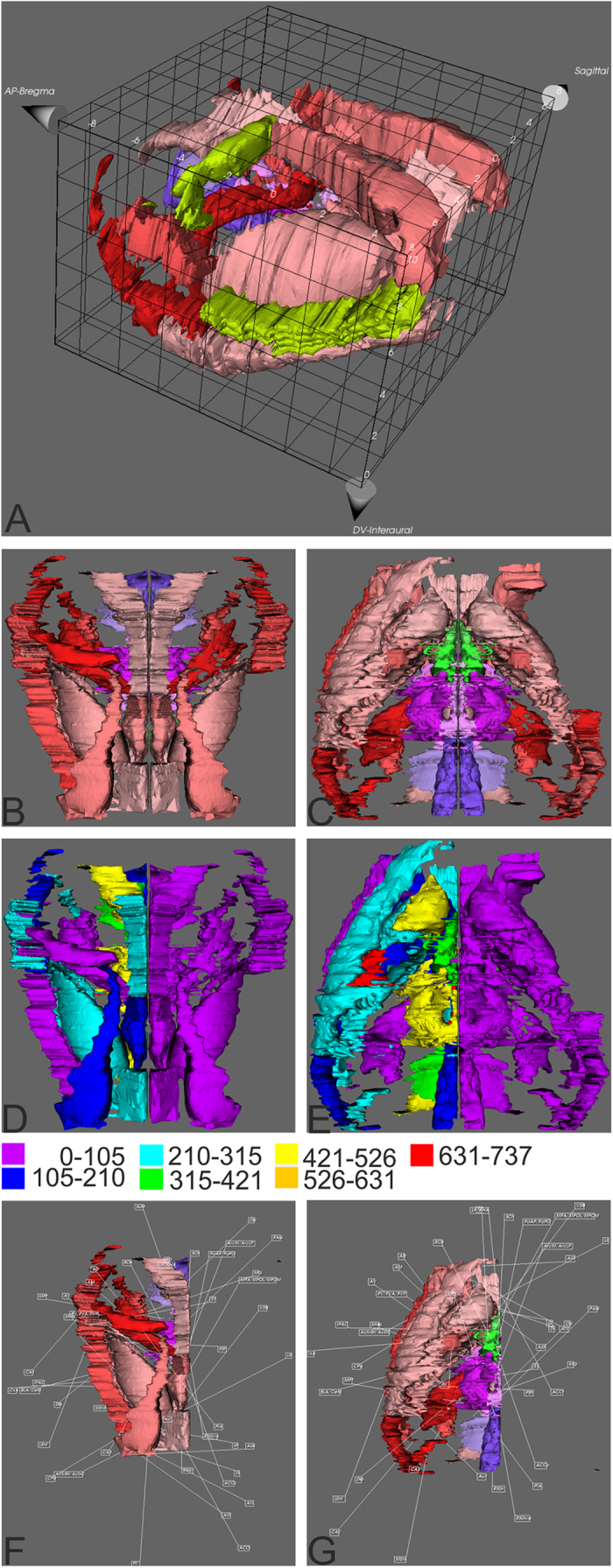
The distribution of regions with changes in Fos expression is topographically discontinuous. Brain regions damaged by stroke (light green) or with changes in Fos expression are visualized in space in 3D (**A**), dorsal (**B**) and ventral (**C**) views. Regions showing Fos expression in stroke rats exploring a novel environment are expressed in colour to reflect the extent of activation in dorsal (**D**) and ventral (**E**) views and the scales are shown below. Regions of interest from the intact hemisphere are labelled with abbreviated anatomical names in dorsal (**F**) and ventral (**G**) views. The expanded text for the regions is shown under Supplementary Table 1, the abbreviation section. The colour codes for Fos regions in (**A**,**B**,**C**,**F** and **G**) are based on neuroontology as defined in neuroVIISAS.

**Figure 2:**
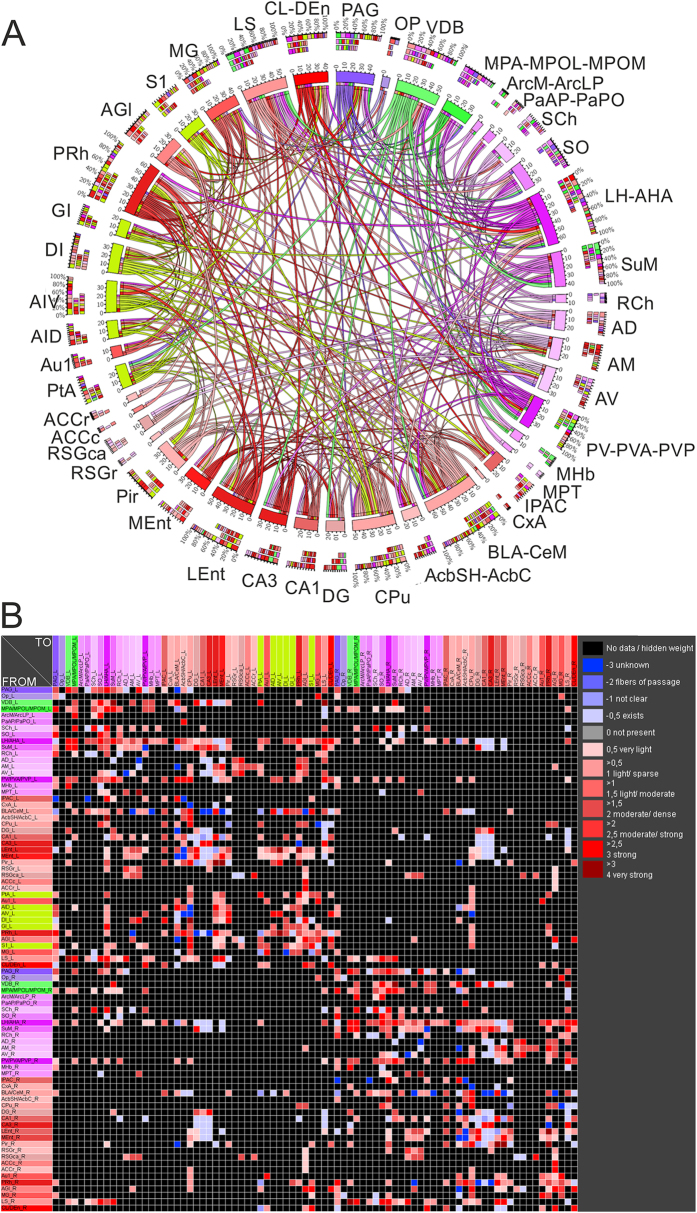
Brain regions damaged by ischemic stroke exhibit relatively high connectivity with regions functionally activated during spatial exploration. (**A**) The chord diagram of weighted connections with above moderate-level of strength (≥1.5) within the Fos network and the six damaged regions in the left hemisphere (light green). Following the removal of weak connections, a massive interconnectivity is still visible within the neural network involved in spatial exploration. Regions with stroke lesion are also well connected with those in the Fos network. Outer arc: relative frequency of inputs and outputs to a particular region. middle arc: “Input arc” or fractions of inputs, Inner arc: “Output arc” or fractions of outputs. (**B**) Adjacency matrix of regions with Fos changes in the bilateral hemispheres and the stroke damaged regions in the left hemisphere. Rows are outputs and columns are inputs. The colour coding of regions is defined within the neuroontology. Shades of a particular colour encode subregions of a parent region.

Constitutive Fos expression was detected in 38 brain regions in awake, sham-operated rats living in their home cages. Many brain regions including supraoptic nucleus, paraventricular thalamic nucleus and claustrum displayed a relatively high number of Fos-immunoreactive cells constitutively, suggesting on-going information processing during the resting state. Spatial exploration increased the expression of Fos in the sham and dMCAO animals by nearly 3- and 5.5-folds, respectively (Supplementary Tables 2 and 3). The mean intensities of Fos expression for all experimental groups are listed by region in Table 1. The spatial relationship among Fos-expressing regions as well as ischemic infarct regions is shown via the interactive 3-D visualization of neuroVIISAS (Fig. 1). The degree of connectiveness between regions is illustrated in the chord diagram using CIRCOS (http://circos.ca/) showing the number of inputs and outputs between any two given regions (Fig. 2A). The hierarchical relationship and weighted connections among the Fos-expressing brain regions are shown in the adjacency matrix (Fig. 2B) according to neuroontology.

Compared to those remaining in the home cages, sham rats exploring a novel environment exhibited activation in most of the 38 regions except for suprachiasmatic nucleus, supraoptic nucleus, interstitial nucleus of the posterior limb of the anterior commissure, basolateral amygdaloid nucleus, medial habenular nucleus, anterodorsal thalamic nucleus and retrochiasmatic area, suggesting that these regions are not specifically involved in processing spatial information.

### Stroke induces region-specific Fos hypoactivation

**Figure 3:**
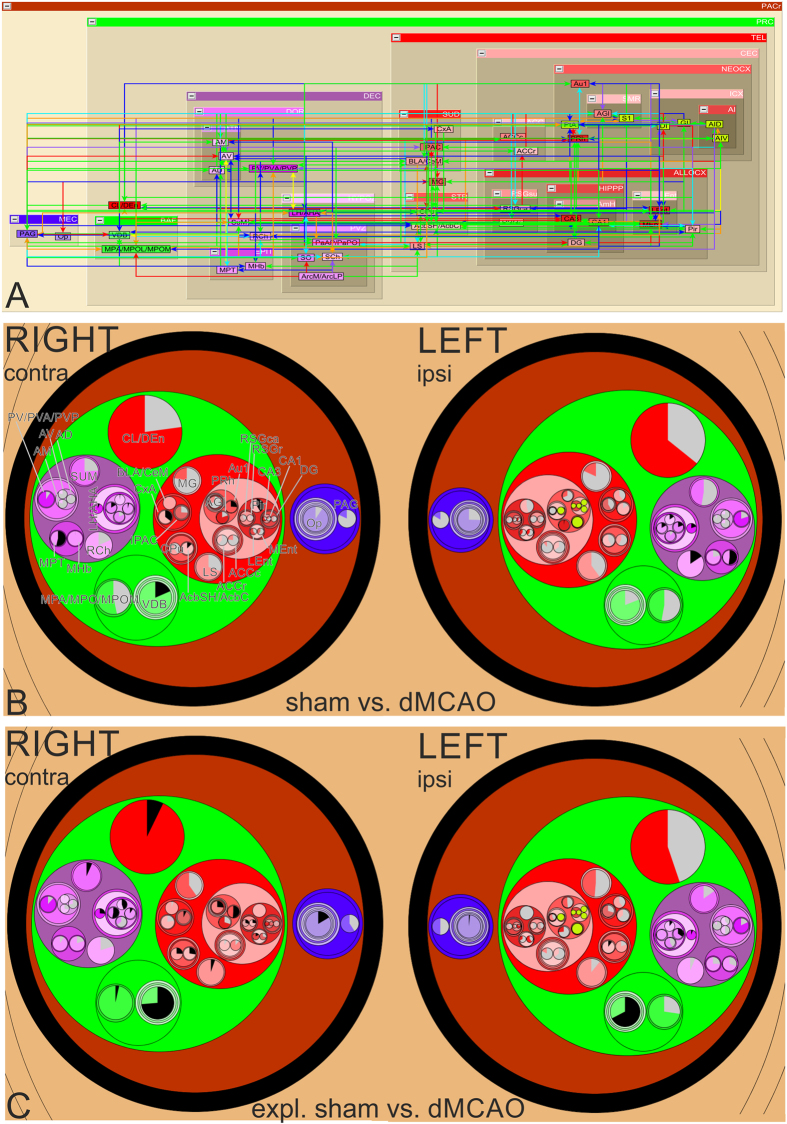
Representation of reactive Fos expression in an orthogonal network and nested circular layout. (**A**) The left hemispheric connectivity of regions with Fos changes in combination with the 6 damaged regions (light green) in an edge bundling orthogonal graph layout. (**B** and **C**) Nested circular visualization of regions with relative Fos changes as indicated by the filling of circles for home-cage condition between sham and dMCAO (**B**), and for explored animals between sham and dMCAO (**C**). Light gray filling: relative decrease of Fos expression. Black filling: relative increase of Fos expression. Light green filled circles belong to the lesioned regions.

A significant region-specific reduction in neural activation marked by Fos was observed in the ipsilateral dentate gyrus, caudal part of the anterior cingulate cortex, rostral part of the anterior cingulate cortex, caudal part of the retrosplenial granular cortex, rostral part of the retrosplenial granular cortex, medial entorhinal cortex, lateral entorhinal cortex, anteromedial thalamic nucleus, anteroventral thalamic nucleus and anterodorsal thalamic nucleus during spatial exploration in dMCAO animals compared to sham animals. dMCAO also had a stronger effect on the reduction of Fos in the explored group compared to the home group in the ipsilateral primary auditory cortex, piriform cortex, anteromedial thalamic cortex, lateral entorhinal cortex and perirhinal cortex. Changes of Fos expression under home-cage and spatial exploration and the effect of stroke are shown in the edge-bundling orthogonal graph layout (Fig. 3A), and in nested circular visualization of regions with relative changes (Fig. 3B and C). Relative difference in Fos listed by region in both groups is shown in Supplementary Table 4.

Five brain regions exhibited significant interaction between the effects of stroke and exploration, including piriform cortex, perirhinal cortex, rostral part of the anterior cingulate cortex, caudal part of the anterior cingulate cortex, and transition region of the amygdala cortex, suggesting that these remote brain regions were specifically affected by stroke and became less engaged during spatial information processing. The extent of hypoactivation marked by Fos is greater in the lesioned hemisphere in comparison with that in the homotopic contralateral regions by 3-folds.

Some brain regions were activated by exploration but were not affected by dMCAO, including the optic nerve layer of the superior colliculus, medial pretectal nucleus, accumbens nucleus, lateral septum, paraventricular hypothalamic nucleus, arcuate nucleus, retrochiasmatic area, lateral hypothalamus, medial preoptic area, supramammillary nucleus, paraventricular thalamic nucleus, anteromedial thalamic nucleus, and anteroventral thalamic nucleus. In contrast, 17 regions in the contralesional hemisphere of rats among dMCAO exploration group showed an increase of Fos expression with regard to the homologue regions in the ipsilesional hemisphere, implicating a compensatory mechanism. Among those regions, contralesional caudate-putamen and paraventricular hypothalamic nucleus may be robustly recruited during exploration in the dMCAO rats likely due to the numerous interconnections between these regions with hippocampal, entorhinal, and neocortical motoric regions (Supplementary Fig. 1). The contralesional primary somatosensory cortex showed the greatest variation coefficient in functional activation among stroke individuals, compared to any other Fos-expressing brain regions including the remaining damaged regions: agranular insular cortex, dysgranular insular cortex, granular insular cortex, and parietal association cortex (Supplementary Table 5).

#### Analysis of the network of Fos-expressing regions and damaged regions

Independent of the expression of Fos, local- or region-specific network parameters have been computed in order to determine relative importance of each region with regard to the connectivity within the network. The bilateral network of Fos-expressing regions and the ipsilateral-damaged regions form a small world yet a relatively highly connected network (Small-worldness = 2.293), consisting of 82 regions and 1215 connections with an average pathlength of 2.083 and an average cluster coefficient of 0.466. In comparison, a randomized network with the same number of regions and edges, albeit with random connections, would only have an average pathlength and cluster coefficient of 1.87 and 0.183, respectively. Apart from these vastly different global network parameters, the Fos-expressing network also has greater reciprocal connections comparing to the randomized network (355 vs. 117), suggesting that the former has a characteristic connecting architecture that cannot be rebuilt by random processes.

The three most connected regions within this network are the lateral and anterior hypothalamus (LH-AHA), basolateral/central amygdala, and periaqueductal gray with output/input connections of 49/37, 35/27, and 24/33, respectively. However, the contribution of a local region to global network properties is correlated with not only the number of connections, but also is defined through local network parameters such as centrality, hubness, and authoritativeness, or through global importance by the Shapley value, for instance. LH-AHA, paraventricular hypothalamic nucleus and supramammillary nucleus are the three regions with the largest eigenvector centrality values (1, 0.741, 0.671). The ranking of regions with regard to subgraph centrality is LH-AHA, lateral septum, and paraventricular hypothalamic nucleus. LH-AHA, basolateral amygdaloid nucleus, and paraventricular hypothalamic nucleus are the regions with the largest hubness parameter and the lateral septum, LH-AHA, and caudate-putamen have the largest authoritativeness (Supplementary Table 6). With respect to the global importance, LH-AHA, nucleus of the vertical limb of the diagonal band, and left primary motor cortex ranked as the top three regions. Overall, LH-AHA has the highest value for all the above network parameters and also the largest vulnerability value (Supplementary Table 7).

#### Principal component analysis (PCA) of the regions with Fos changes and damaged regions

**Figure 4:**
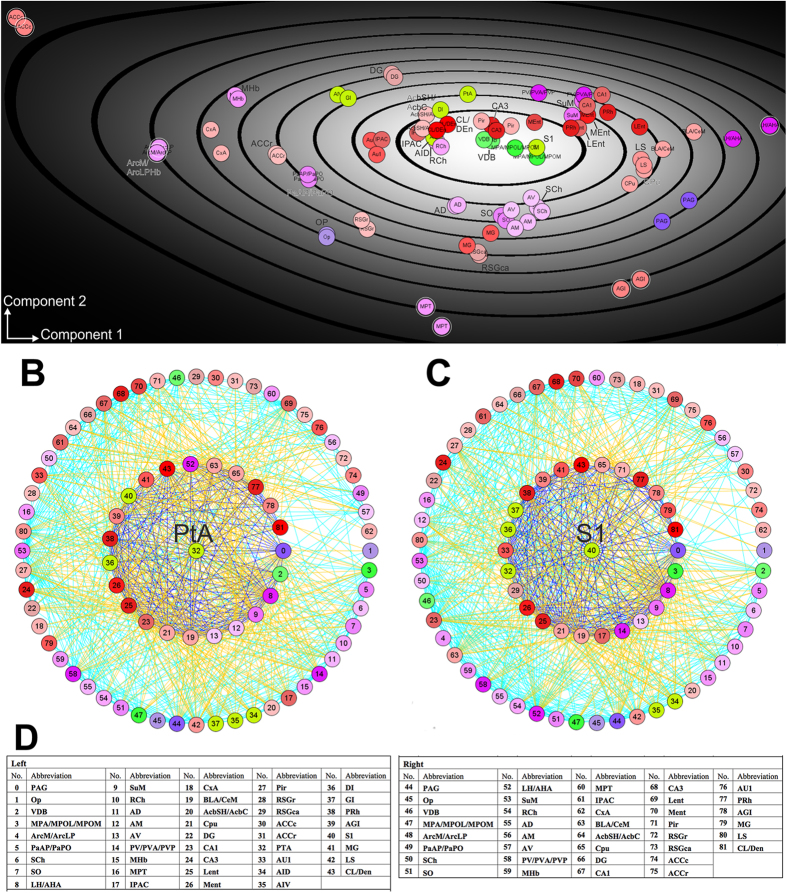
Principal component analysis (PCA) of damaged regions and regions with Fos changes reveal differences in connection patterns. Six local parameters (DG_All_, CLu_All_, CluC_2_, AvgDG_nb_, VC_DG_, Loc) were applied to PCA. The bilateral network of regions with Fos changes and the 6 damaged regions in the left hemisphere were analysed. (**A**) Gray tones in the PCA diagram indicates the density of regions within an encircled area of the PCA plane. The component 1 in the PCA diagram is the X-axis that corresponds essentially to a linear combination of the parameters degree all, locality and with a negative influence of the cluster coefficient, i.e. X = (degree all + locality) – cluster coefficient. These three parameters are highly correlated among themselves, meaning if a region has a high degree all it probably also has a high locality and a small cluster coefficient. The variation coefficient of the neighbour degree mainly determines the second component. B-C, The direct and indirect connectivity relationship of ipsilateral parietal association cortex (PtA: 32) (**B**), and primary somatosensory cortex (S1: 40) (**C**). In the center of the circular visualization is the region of interest. Around this center region are its direct neighbours or directly connected regions shown as the inner circle, surrounded by the indirect neighbours as the outer circle. Regions are encoded in the circular diagrams and listed in (**D**). Damaged regions in the left hemisphere are shown in light green. Regarding the colour code for the connecting lines, red or black represents connections from center to inner ring regions. Blue: connection among inner ring regions. Yellow: connections from inner to outer ring. Cyan: connection among outer ring regions.

**Figure 5:**
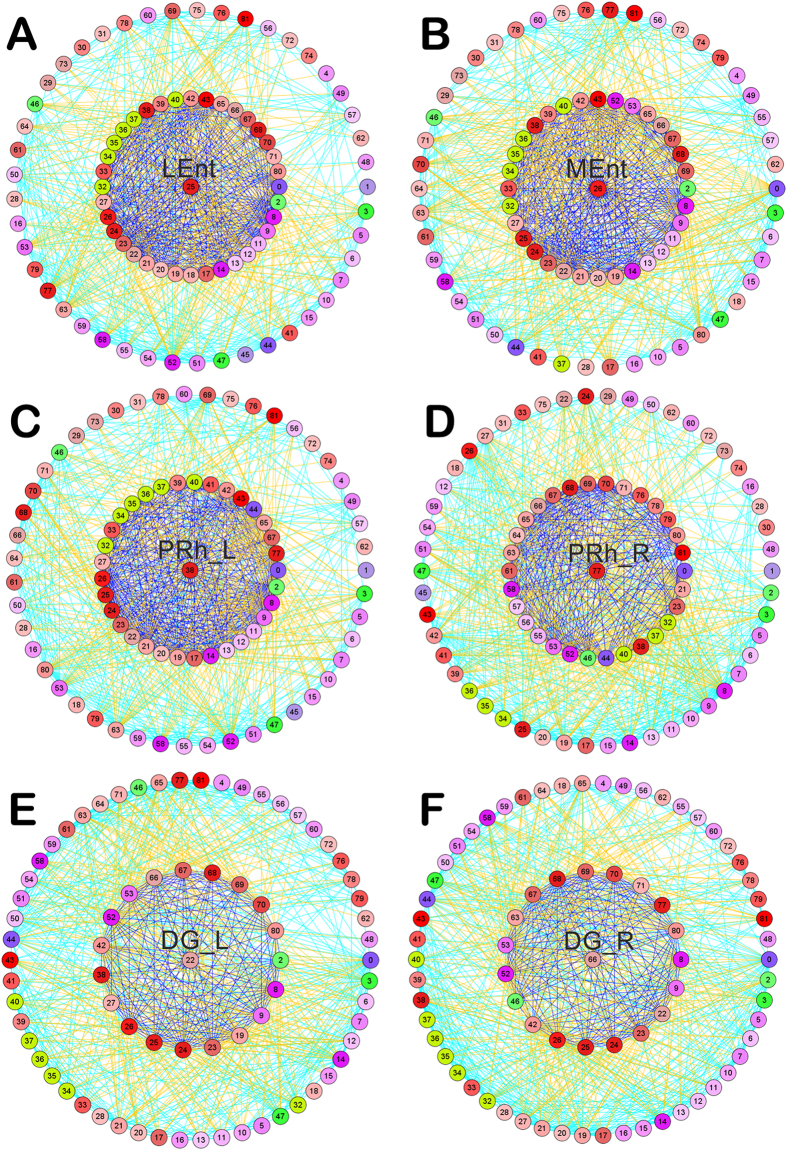
Regions directly connected to the stroke lesion have more connections compared to those that do not. Connectivity patterns of the lateral entorhinal cortex (Lent: 25) (**A**) and medial entorhinal cortex (MEnt: 26) (**B**), ipsilesional (PRh_L: 38) (**C**), contralesional perirhinal cortex (PRh_R: 77) (**D**), ipsilesional (DG_L: 22) (**E**) and contralesional dentate gyrus (DG_R: 66) (**F**). Similar to Fig. 4, the region of interest is placed in the center of the circular visualization, surrounded by its direct neighbours or directly connected regions shown as the inner circle, then by the indirect neighbours as the outer circle. Regions are encoded in the circular diagrams and listed in Fig. 4D. Damaged regions in the left hemisphere are shown in light green. Connectivity of the LEnt with direct connections to damaged regions has a strong interconnectedness with the directly connected regions, in contrast to the weak interconnectedness observed for DG_L or DG_R, neither of which has a direct connection with the damaged regions. Colour code for the connecting lines: red or black represents connections from center to inner ring regions. Blue: connection among inner ring regions. Yellow: connections from inner to outer ring. Cyan: connection among outer ring regions.

A PCA has been conducted to determine the relationship of local connectivity with the six local network parameters, i.e. degree all, cluster coefficient all, cluster coefficient of second neighbours, average degree of neighbours, variability coefficient of the degree and locality^12^. The PCA rotates the 6-dimensional data in such a way that the first two dimensions contain as much of the variance of the data as possible. These two dimensions of the regions of the bilateral network are shown in Fig. 4A. Not surprisingly LH-AHA has the largest degree all values and is located towards the right in the PCA plane that corresponds to a smaller cluster coefficient. In contrast, the anterior cingulate cortex caudal part (ACCc) has the smallest degree all value placing it at the lower left corner of the PCA plane. The damaged regions are located relatively close together compared to other regions that are distributed over the entire PCA plane, consistent with the fact that the local parameters of these six regions are similar. Regions that are directly connected with the damaged regions have 268.66 connections, compared to 116.93 connections of those without direct connections to damaged region. Examples of regions that are directly connected with damaged regions are shown in circle diagrams (Figs 4 and 5). These regions are also located relatively close together in the PCA-plane.

To illustrate the connectivity of regions of interest (ROIs) with regard to the first and second neighbours, we have selected a few examples of damaged (Fig. 4B and C) and undamaged regions (Fig. 5). In general, there are fewer immediate neighbours and lower density of connections to the damaged region (PtA or S1) in contrast to the limbic regions that are close to the lesion such as the entorhinal cortex or perirhinal cortex. In contrast to the lateral entorhinal cortex, which has direct connections to all damaged regions, the medial entorhinal cortex does not directly connect to the damaged granular insular cortex (Fig. 5A and B). A connection from the medial entorhinal cortex to the granular insular cortex is possible going through the intermediate region LH/AHA. On the other hand, the contralateral perirhinal cortex has direct connections to ipsilateral damaged regions, namely the primary somatosensory cortex, granular insular cortex and parietal association cortex. Furthermore, the ipsilateral perirhinal cortex also has direct connections to all ipsilateral damaged regions (Fig. 5C and D). In contrast to the entorhinal cortex, the dentate gyrus is not directly connected to the damaged regions and exhibits weaker connections with it first neighbor, although it is a major input region of the hippocampal formation (trisynaptic circuit) and harbours neuronal progenitor cells that are necessary for post-stroke functional recovery^23^. Despite lacking a direct ipsilateral or contralateral connection with damaged regions (Fig. 5E and F), dMCAO chronically reduced the synaptic plasticity in the perforant pathway^23^, possibly by changing the input from the damaged regions to the lateral entorhinal cortex, by indirectly affecting the hippocampus.

#### Correlation analysis of network parameters and Fos expression

We next determined whether stroke-induced functional impairment as reflected by differential reduction in Fos expression in various brain regions can be predicted by the known connectivity relationship among these regions. Instead of analysing the complete bilateral connectome of the rat nervous system, which generated little correlation (data not shown), we conducted the analysis in the partial connectome solely consisting of regions expressing Fos (as in Fig. 2). The correlation coefficient by Pearson is a measure of the strength of the linear relationship between two variables and has values between -1 and 1, which depict a perfect negative and positive correlation, respectively. A total of 43 network parameters were examined with regard to their correlation with the Fos-expression changes for both weighted and unweighted connections excluding the damaged regions, i.e. agranular insular cortex, dysgranular insular cortex, granular insular cortex, primary somatosensory cortex, and parietal association cortex. For the home-caged animals, the network parameters in Subgraph Centrality (SC), Eigenvector Centrality (EC), Hubness, and Authoritativeness of these ipsilateral regions receiving direct input from the dMCAO-damaged regions is modestly correlated with Fos-expression level with correlation coefficients of 0.61, 0.54, 0.51 and 0.41, respectively (Supplementary Fig. 2). The results suggest that the level of Fos expression appears to be weakly correlated with the importance of a region in our connectome framework. However, due to the greater variation in Fos level among the animals exploring a novel environment, the correlation coefficients were further reduced in those groups. Our connectome modelling data suggest that the current data set in Fos mapping cannot provide a robust prediction of functional activation patterns based on the connectivity information provided by neuroVIISAS.

**Figure 6:**
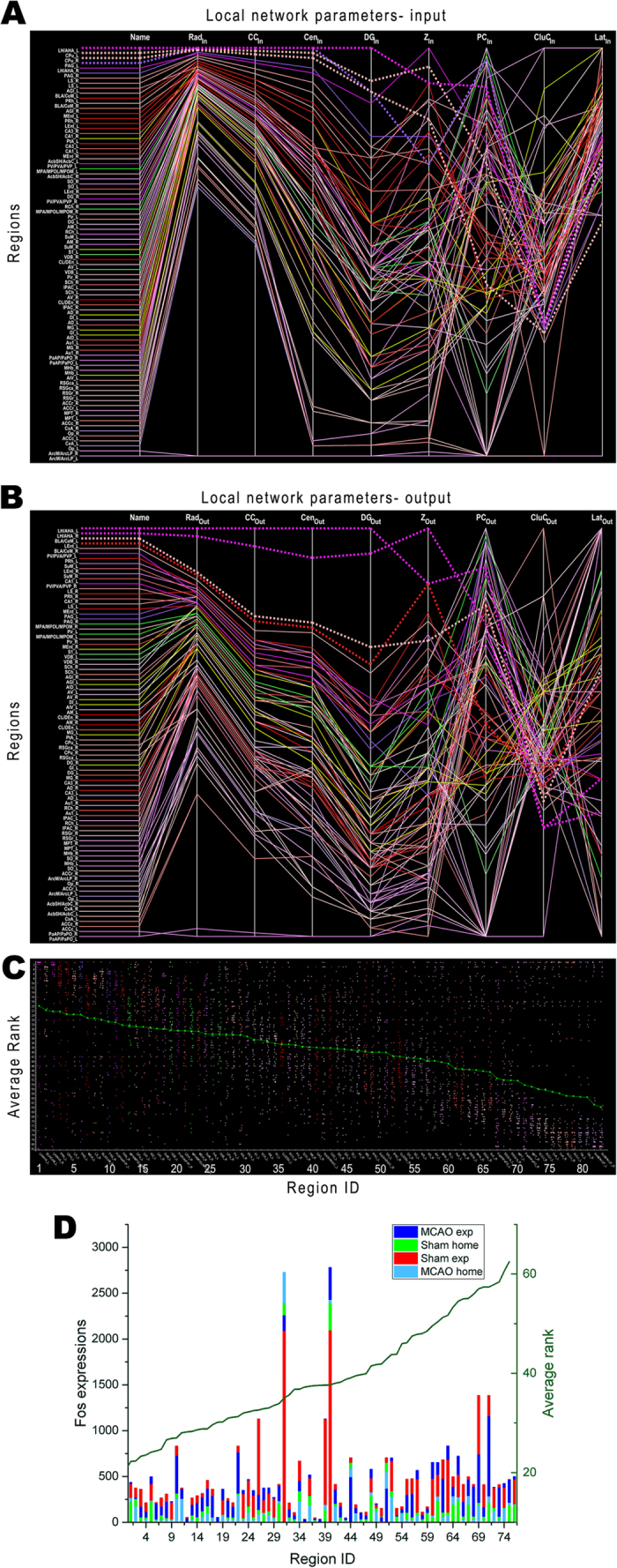
Ranking of local network parameters and Fos expression. The similarity of local parameters (X-axis) over Fos-expressing regions (Y-axis) is demonstrated in the parallel coordinate representation. The local network parameters are grouped according to input (**A**) and output (**B**) diagrams. Dashed lines indicate regions with largest degree inputs and outputs, respectively. The input as well as output representations are arranged independently regarding the similarity of parameters. (**C**) Rank analysis of Fos-expressing regions in network parameters. The local parameters have been sorted for similarity. The mean ranks were calculated and sorted for each region. The regions (X-axis) with highest ranks are near the ordinate (Average rank). The green curve indicates the mean rank of all parameters for each region. Dots in (**B**) are colour coded according to neuroontology. (**D**) The relationship between Fos expressions and average ranks (dark green curve) over regions in the network. A greater similarity in Fos level in a given region is shared within a behavioural group (home-cage or exploration), rather than within a procedure group (sham or MCAO). The level of neuronal activation as marked by Fos appears to be region-dependent, and bears no relationship with the average rank of that region in the network. X-axis numbers and abbreviations: 1:LH/AHA_L, 2: BLA/CeM_L, 3: LH/AHA_R, 4: PRh_L, 5: PV/PVA/PVP_L, 6: LEnt_L, 7: PAG_L, 8: LS_L, 9: MEnt_L, 10: BLA/CeM_R, 11: MPA/MPOL/MPOM_L, 12: LS_R, 13: CPu_L, 14: PV/PVA/PVP_R, 15: PAG_R, 16: PRh_R, 17: AGl_L, 18: CA1_L, 19: SCh_L, 20: VDB_L, 21: MPA/MPOL/MPOM_R, 22: SuM_L, 23: Pir_L, 24: MEnt_R, 25: RCh_L, 26: CA1_R, 27: CPu_R, 28: LEnt_R, 29: VDB_R, 30: AGl_R, 31: SuM_R, 32: PtA_L, 33: SCh_R, 34: CL/DEn_L, 35: AcbSH/AcbC_L, 36: Pir_R, 37: SO_L, 38: S1_L, 39: RCh_R, 40: AM_L, 41: AV_L, 42: AcbSH/AcbC_R, 43: IPAC_L, 44: SO_R, 45: CA3_L, 46: DG_L, 47: AM_R, 48: CA3_R, 49: CL/DEn_R, 50: DG_R, 51: DI_L, 52: IPAC_R, 53: AV_R, 54: MG_L, 55: AD_L, 56: MHb_L, 57: AID_L, 58: AD_R, 59: AIV_L, 60: RSGr_L, 61: Au1_L, 62: RSGr_R, 63: PaAP/PaPO_L, 64: GI_L, 65: Au1_R, 66: MHb_R, 67: RSGca_L, 68: MPT_L, 69: RSGca_R, 70: PaAP/PaPO_R, 71: MG_R, 72: MPT_R, 73: CxA_L, 74: ACCr_L, 75: CxA_R, 76: ACCr_R, 77: Op_L, 78: Op_R, 79: ACCc_L, 80: ACCc_R, 81: ArcM/ArcLP_L, 82: ArcM/ArcLP_R.

We then determined the relationship between Fos expression and the importance of a given region in the network by ranking the local input and output network parameters. LH/AHA, CPu and PAG have relative large values of the input parameters radiality (Rad_in_), closeness centrality (CC_in_), centroid (Cen_in_) and the degree (DG_in_) (Fig. 6A). In contrast, the similarity of the parameters z-score (Z_in_), participation coefficient (PC_in_), cluster coefficient (Clu_in_) and laterality (Lat_in_) is weaker, providing more crossings of lines of parameter expressions. The same output parameters have the largest values for LH/AHA of the ipsilateral lesioned and contralateral intact hemisphere (Fig. 6B), suggesting that LH/AHA-efferents contribute to a strong proportion of network structure. However, CPu and PAG do not have as large values as for the local parameter afferents in Fig. 6A. Instead of the CPu, the regions ipsilateral BLA/CeM and LEnt have abundant efferents. Consistent with the degree of inputs and outputs, ipsilateral LH/AHA, BLA/CeM, contralateral LH/AHA and PRh have the highest mean ranks with regard to local network parameters (Fig. 6C), reflecting their great importance in the network. In the parallel coordinate representation, the Fos values of the four groups of animals are similar among themselves, which are comparable with the similarity of their local parameters (Fig. 6, Supplementary Fig. 3). The analysis suggests that the absolute Fos expression does not correlate with the importance or mean rank of a given region in the network, although it generally coincides within the home cage or exploration group, respectively (Fig. 6D). However, two exceptions are noticed in Pir and CxA, both expressing much reduced Fos level in the stroke compared to non-stroke animals in the exploration group. Without direct connection between each other, CxA and Pir have reciprocal connections via VDB (CxA ↔ VDB ↔ Pir), a region with several connections to hypothalamus, thalamus and hippocampus and expressing surprisingly more Fos in both hemispheres in the MCAO exploration group compared to the sham exploration group. The Pir-hippocampal interconnectivity also coincides with the hypoactivation of the latter during exploration in the MCAO group, as well as spatial memory impairment revealed by the Barnes maze test^23^.

#### Correlation analysis of spatial distances and Fos expressions

**Figure 7:**
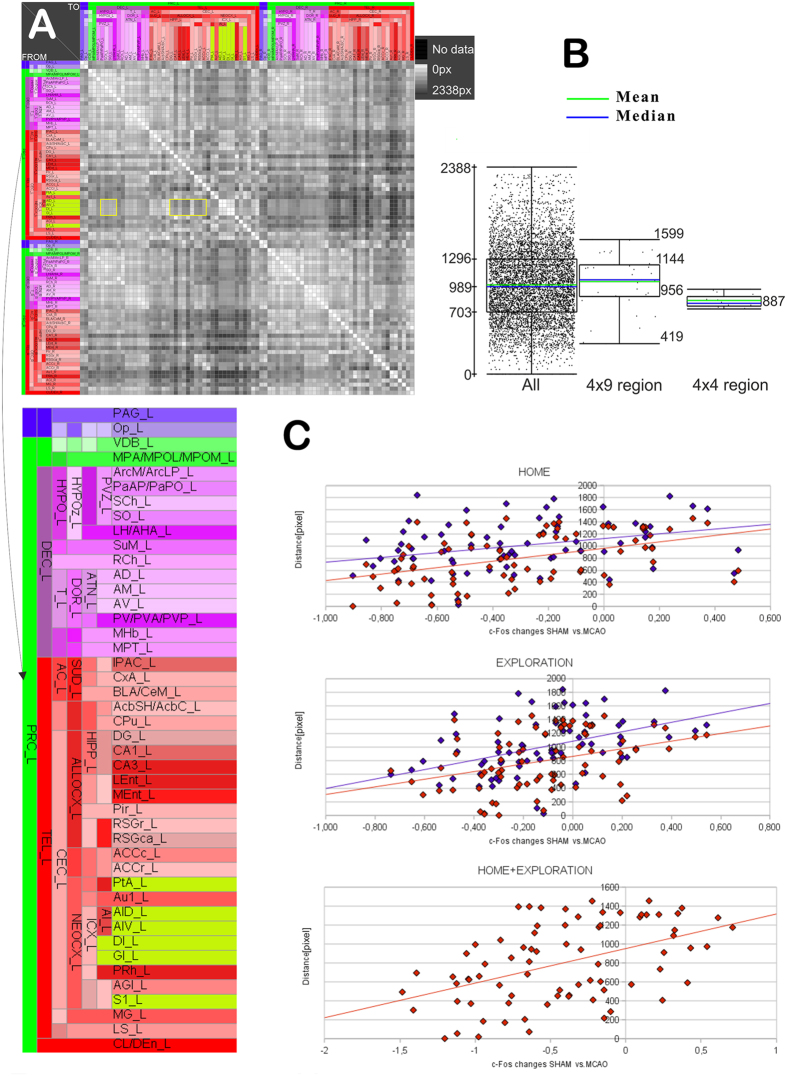
The relationship between spatial distances of regions of interest and Fos expression. (**A**) Spatial distance matrix between regions with Fos changes and damaged regions. The distances between some damaged regions to the diencephalic brain regions (yellow square, 4 × 4) are smaller (gray region labels) and to the cortical regions (yellow rectangle, 4 × 9) (darker gray values) than those to the remaining regions. (**B**) Distribution of spatial frequencies of the whole matrix and comparison (t-test) of average distances of some cortical and diencephalic regions with the damaged regions. The spatial distances are significantly larger between cortical and the damaged region (p < 0.05), compared to those between diencephalic and the damaged regions (p < 0.001). (**C**) Correlations of parietal association cortex (red) and primary somatosensory cortex (blue) with the Fos expression values of regions that show Fos changes.

Based on the concept of “Euclidean distances of centres of gravity of region”, we determined how the spatial distance between a particular brain region and the region of stroke injury affects the extent of functional activation (Fig. 7A). Largest correlations were found for the distances to the parietal association and primary somatosensory cortex as shown in Fig. 7 and Supplementary Table 3, indicating that Fos-expression level of a particular region is highly influenced by the spatial distance to the damaged regions (Fig. 7B). In addition, such correlations are larger on the ipsilateral side. Interestingly, animals exploring the spatial environment have larger correlation values than those of the home-caged ones. Our data suggest that spatial proximity to ischemic infarct appears to be a relevant factor in predicting the extent of functional activation in a given non-damaged region in the context of connectivity.

## Discussion

Emerging evidence from structural connectivity analyses suggests that stroke leads to changes in brain activity^24^, reorganization^25^ and structure^26^. To understand how cortical stroke differentially affects functional activation in various brain regions, we performed a thorough brain mapping study via Fos-imaging comparing stroke and non-stroke rats exploring a novel spatial environment. We found that a total of 38 brain regions were activated following spatial exploration. Cortical stroke reduced the activation in some selective brain regions of the ipsilateral side, whereas increased the activation in only few regions in hemisphere contralateral to the lesion^27^, pointing to a compensatory mechanism or disinhibition. The connectivity matrix shows connections between the six dMCAO-lesioned regions and the contralateral homotopic regions, although it is unclear how the reduced inputs to the contralateral regions affect their functional activity. We also performed a connectome modelling analysis using the neuroVIISAS-framework to determine whether remote regions affected by stroke could be predicted by known information in connectivity among these regions and whether the degree of functional impairment is correlated with the distance to the infarct location. To the best of our knowledge, this is the first study set to understand how stroke affects functional activation based on structural connectivity information using a connectome platform. This is also the first *in silico* approach to explore the possibility of predicting brain regions suffering from functional impairment after ischemic stroke.

Expression of the immediate early gene product Fos is an indirect correlate of increased neuronal activity^28^,^29^,^30^ and has repeatedly been shown to be induced under conditions of learning^5^,^6^,^31^,^32^,^33^. Fos expression patterns in the rodent brains are similar to those identified using 2-deoxyglucose method (2DG) in rhesus monkeys performing spatial working-memory task^34^. However, the 2DG technique has a low sensitivity and it does not distinguish metabolic changes between neurons from glia^35^, which can potentially reduce the specificity of connectome and network structure. Fos and Zif268 are not only required for LTP and long-term memory^36^,^37^,^38^,^39^ but also been used extensively to map memory activity (both recent and remote memory) using spatial memory task^10^,^40^,^41^. Changes in Fos-expression patterns have been detected in animals performing spatial learning task following lesions of the hippocampus or structures connected to the hippocampus such as anterior thalamic nuclei and fornix^6^,^7^,^42^,^43^. In fact, Fos-imaging studies have contributed strong evidence to suggest that the thalamus, hippocampus, and parahippocampal cortices form the key components of the interdependent neuronal network involved in spatial mnemonic processing^43^,^44^,^45^,^46^,^47^,^48^. Consistent with our current knowledge in the pattern of brain activation, the paradigm of spatial exploration used in our study requires multiple brain regions to work together for the recognition of a novel object in a novel spatial configuration^49^. However, the main caveat in using Fos imaging to map functional activation is that not all neurons are capable of expressing Fos. Satb1, one of the known limiting factors required for proper temporal dynamics of immediate-early gene expression^50^, has a restricted neuronal expression pattern in the adult mouse brain^51^. The second limiting factor is the sensitivity of immunostaining. Some small regions with very low levels of Fos expression could not be evaluated, which may or may not be attributed to lacking Satb1 expression; however, these regions may play regulatory roles and could be relevant for network analysis and predictive issues. In comparison, mapping neuronal activity by quantification of regional cerebral blood flow using ^14^C-iodoantipyrine autoradiography would allow functional *in vivo* measurement in all brain regions^9^, although with the added disadvantage of involving a radioisotope. Despite the unrestricted nature of the blood flow data with respect to brain regions, the relatively low resolution of this *in vivo* imaging method offers little distinction between structures at the level of cortical and subcortical regions, which may interfere with the connectome analysis. Nonetheless, by correlating spatial distances between damaged regions and regions with Fos changes, we found that the parietal association cortex and primary somatosensory cortex had the greatest positive correlations ipsilaterally. It suggests that Fos expression might be influenced or determined by at least the spatial relation to the ischemic lesion. The inverse relationship between spatial distance and the extent of functional hypoactivation in our study is in line with a recent report demonstrating that the disruption of neural activity in the barrel cortex surrounding photothrombotic microinfarcts appeared to be inversely correlated with the distance from the infarct border, resulting in a greater volume of neural deficits compared to the volume of infarct core by 12 folds^52^.

The global parameter average pathlength indicates the average distance in a network of a given region to any other region. A value of 1 means all regions are completely connected among themselves. A relatively large average pathlength in a neuronal network (e.g. >3) suggests that the signals transported through the network must pass more intermediate nodes before reaching a target region. A small-world network can be characterized as follows: If the majority of nodes in a network are not neighbours of one another and the neighbours of any given node are likely to be directly interconnected with each other, such network qualifies as a small-world network, in which most nodes can be reached from every other node by a small number of steps. The small-worldness is a further global network parameter that quantifies this feature by comparing a given network with randomized networks. Should a network have a small-world structure, the small-worldness would be larger than 1. The bilateral network of Fos-expressing regions has a relatively large small-worldness parameter of >2, indicating a strong tendency of the small-world phenomenon. The clustering coefficient indicates the proximity between the neighbours of a region within a clique or a complete graph. The average clustering coefficient is the average over all local cluster coefficients. Large average clustering coefficients are typical for small-world networks and small coefficients are typical for random networks^53^.

By comparing the global network parameters average pathlength, small-worldness and average cluster coefficient of the network that includes the damaged regions and the network without damaged regions, only a small difference is detected. This suggests that the global network structure is weakly affected by removing the damaged regions. However, the largest decrease of 12.3% of a global parameter was found for the number of reciprocal connections in the network without damaged regions. Hence, reciprocity is most strongly influenced by removing the damaged regions. Regions in the entorhinal cortex (Lent and MEnt), perirhinal cortex and temporal lobe are reciprocally connected with the damaged regions, raising the possibility that ischemic injury can impact functional capacity in learning and memory among stroke animals. Alternatively, following the removal of the damaged regions, the activity of the remaining network is altered via changes in synaptic activity or connectivity among themselves via re-organization at the structural and functional level.

By computing the correlation of changes of a network parameter like the degree (inputs and outputs) of a region with its changes of Fos expression, one can decipher whether it is feasible to predict stroke effect on regional functional change on the basis of network parameters^11^,^22^. When the analysis was first performed in the whole connectome, it yielded weak correlation. The most apparent reason attributable for this prediction failure is that regions of the whole connectome that may have regulatory effects on spatial navigation do not show Fos changes during exploration, and hence are missing in the analysis. Second, regions of the whole connectome that have direct and/or circular regulatory connections to regions involved in spatial navigation may not be activated themselves during spatial exploration, but can be activated by other tasks. Third, because the six damaged regions are relatively large and strongly interconnected in the whole connectome (as determined by the sum of inputs and outputs, known as DGa: parietal association cortex: 354; agranular insular cortex dorsal part: 241; agranular insular cortex ventral part: 159; dysgranular insular cortex: 119; granular insular cortex: 147; primary somatosensory cortex: 923), the complexity of effects on the connectivity and changes of network parameters is profound. Fourth, the whole connectome of the rat brain contains additional pathways that bypass functionally established routes of those regions where Fos have been measured, e.g., entorhinal cortex (Ent) → DG → CA3 → CA1 through hippocampal regions that are relevant for spatial navigation. These shortcut routes that partially bypass the trisynaptic hippocampal pathway are direct connections from Ent to CA3, Ent to CA1, and SG to the subiculum. These specific bypassing connections that are missing in the partial connectome of regions with Fos changes could also be responsible for a reduction of correlations. Besides, complex systems are embedded in the mere structural connections; e.g. neurotransmitters/neuromodulators, ion channels, receptors, synaptic/post-synaptic or axonal transduction properties. These molecular and neurophysiological features along with local circuitry (reciprocal connections, local interneuronal connections) may be the missing links for a reliable prediction at the level of a whole connectome. Alternatively, regions with comparable molecular/functional layout similar to the damaged regions may take over functional activity, at least partially, correlated with spatial navigation-induced synaptic plasticity.

The analysis of the partial connectome consisting of the regions expressing Fos uncovered larger correlations of the EC, SC, hubness and authoritativeness for regions of dMCAO than those in the whole connectome described before, although still not sufficiently large for a reliable prediction of the functional state for each brain region. A reasonable explanation for the smaller cc in the exploration group compared to the home cage group is that the former group exhibited a larger variability of brain activation among animals. Alternatively, structural connectivity of the selected regions could be better correlated with Fos at resting state, rather than an activated state. Besides, behaviour induced changes in CNS rewiring sometimes occurs in the spinal cord rather than the forebrain, as demonstrated in a recent study after photothrombotic stroke^54^. It is also conceivable that weighted and directed connectivity patterns could be regulated after ischemic stroke by mechanisms that are not detectable or complementary at the level of Fos expression or mesoconnectomics.

In addition to the correlation of static local network parameters with Fos expression one may correlate dynamic network features derived from mean field theory, neural mass models, diffusion models and neuronal population models of the connectome with regard to regions of interest with Fos expressions. These advanced dynamic analyses are currently under development. Nonetheless, there are limitations regarding the current static connectome analysis. The collation of connections described in publications using monosynaptic retrograde and anterograde tract-tracing techniques allows for the development of connectomes at mesoscale levels^15^,^16^,^17^,^55^. Such a metastudy of the whole (peripheral and central nervous system) rat connectome is a work-in-progress project using the generic neuroVIISAS framework^11^,^12^. So far, about 4500 publications have been evaluated which basically describe tract-tracing results in normal juvenile and adult rats. In addition, publications that describe tract-tracing results as a supporting method are also under investigation. Hence, the substantial tract-tracing literature from 1971, when first results of retrograde HRP transport have been published^56^, to the present has been evaluated and connections have been curated and integrated into the connectome project of the rat nervous system. However, it is difficult to estimate how many connections are not discovered and are missing in the rat connectome project. In addition, connections that have been judged to be uncertain, fibres of passage, or statements that are not clearly described in terms of sources and targets of projections are not taken into account in the connectome investigated here. Furthermore, projections of collaterals and pathways have been split in terms of networks and graphs as singular source-target-connections to consider as much reliable data as possible.

In conclusion, the interrelation of structural connectivity and functional activity at the histological level is complex. Our results suggest that it is possible to estimate the dependency of a region and its Fos expression in relation to the distance from the damaged regions. Relatively small correlation coefficients of particular local network parameters of the whole connectome and Fos-expression values were determined in the network of regions with Fos changes, although modest correlations have been found in the partial connectome. Hitherto, these parameters cannot be reliably used in the analysis of stroke injury to predict the location or extent of functional impairment based on our current set of data. The weak correlation could be partially explained by relative large inter-individual Fos variability. In addition, an increase of the sensitivity of Fos measurement would provide expression levels for additional regions that could be relevant for the connectivity Fos-expression correlation. A clear advantage could be expected from high-resolution tractography of diffusion imaging data, although in principle the MRI method is not feasible for the study of animal cognitive behaviour-associated brain activation. Besides, these data would be non-directed and may not be able to provide an extensive network mapping due to a very large amount of very short connections. Nonetheless, based on correlation analysis it is possible to estimate dependency of a region and its Fos expression in relation to the distance from the damaged regions.

## Additional Information

**How to cite this article:** Schmitt, O. *et al*. Prediction of regional functional impairment following experimental stroke via connectome analysis. *Sci. Rep.* **7**, 46316; doi: 10.1038/srep46316 (2017).

**Publisher's note:** Springer Nature remains neutral with regard to jurisdictional claims in published maps and institutional affiliations.

## Supplementary Material

Supplementary Materials

## Figures and Tables

**Table 1:** The effect of spatial exploration and ischemic stroke on the extent of neuronal activation.

Region	Sham Home	Sham Explor	dMCAO Expl-ips	dMCAO Expl-con	Specific functions	Functions														
						Learn	Mem	Navi	Move	Alert	Spat	Exec	Emo	Resp	Stress	Circa	Theta	Thi	Hung	Therm
AcbSH/AcbC	290.8	481.3	517.8	578.9	Reward, pleasure, addiction, aggression, fear															
ACCc	63.2	410.7	161.6	375.7		+	+					+	+	+						
ACCr	64.0	415.6	219.2	387.3		+	+					+	+	+						
AD	64.5	153.2	53.2	71.4		+	+			+										
AGl	58.2	291.0	204.0	347.3					+											
AM	55.9	213.0	136.8	169.2		+	+			+										
ArcM/ArcLP	187.1	467.5	461.7	500.0	Hormone release															
Au1	55.9	476.3	305.8	655.1																
AV	34.7	105.9	75.4	101.9		+	+			+										
BLA/CeM	257.1	374.0	265.2	361.4			+						+							
CA1	5.2	56.8	38.3	54.5		+	+				+									
CA3	4.9	34.8	30.7	33.2		+	+				+									
CL/DEn	340.3	667.8	447.7	703.2			+													
CPu	121.7	317.1	271.3	376.5		+			+											
cRSP	109.3	683.3	471.2	833.6																
CxA	213.8	1385.1	736.9	1155.6	Amygdala-Cortical connectivity															
DG	25.5	48.9	36.3	45.5		+	+				+									
LEnt	53.2	213.6	109.8	167.4			+	+												
LH/AHA	222.1	417.2	438.2	497.9												+		+	+	+
LS	32.4	217.3	200.2	225.9																
MEnt	49.5	309.8	219.5	349.5			+	+												
MG	34.4	112.1	69.5	78.8																
MHb	105.3	170.3	138.8	145.5	Reward, pain, nutrition					+					+	+				
MPA/MPOL/MPOM	177.3	456.6	364.6	468.4	Male copulation behav.													+		+
MPT	194.4	497.5	385.4	460.8	Regulates pupillar light reflex															
Op	159.3	378.0	382.6	424.0	Behav., retina-V1-connection															
PaAP/PaPO	237.4	519.1	652.2	721.8	Appetite, autonomic functions, regulates pituitar															
PAG	77.9	266.2	167.8	186.1	Pain, defensive behav., female copulation behav.															
Pir	136.4	1128.3	296.7	1128.4	Olfaction															
PRh	55.6	362.8	166.6	282.9	Recognition environmental stimuli		+													
PV/PVA/PVP	316.2	832.2	722.0	761.0	Arousal, energy balance, salt appetite										+	+				
RCh	203.8	413.1	393.5	351.5												+		+	+	+
RSGr	107.6	472.0	295.3	566.4	Recall episoidc information															
SuM	56.3	260.2	231.3	272.8							+						+			
VDB	84.6	242.5	363.1	374.2													+			
SO	650.5	703.1	490.1	365.9	Regulates pituitary, anti-diuretic															
Sch	2390.7	2091.3	2255.4	2781.4																
IPAC	156.4	193.6	151.4	204.0	Amygdala, hypothalamic connections															

Mean intensities of Fos expressing regions were shown by region in 4 experimental groups including the sham home cage group (N = 11), the sham exploration group (N = 8), the dMCAO exploration group (N = 10) with ipsilateral Fos expression (Explips), and the dMCAO exploration group with contralateral Fos expression (Explcon). The specific function of a given region is also indicated on the right columns. Abbreviations: Learn: Learning, Mem: Memory, Navi: Navigation, Move: Movement, Alert: Modulation of alertness, Spat: Spatial processing, Exec: Executive functions, Emo: Emotion formation, Resp: Respiratory control, Stress: Stress processing, Circa: Circadian cycle and sleep/wake cycle control, Theta: Generation of theta waves, Thi: Thirst, Hung: Hunger, Therm: Thermoregulation, Behav: Behaviour.

## References

[b1] Bederson J. B. . Rat middle cerebral artery occlusion: evaluation of the model and development of a neurologic examination. Stroke; a journal of cerebral circulation 17, 472–476 (1986). 10.1161/01.str.17.3.472 3715945

[b2] Iizuka H., Sakatani K. & Young W. Selective cortical neuronal damage after middle cerebral artery occlusion in rats. Stroke; a journal of cerebral circulation 20, 1516–1523 (1989). 10.1161/01.str.20.11.1516 2479127

[b3] Liu F. & McCullough L. D. Middle cerebral artery occlusion model in rodents: methods and potential pitfalls. J Biomed Biotechnol 2011, 464701, doi: 10.1155/2011/464701 (2011). 21331357 PMC3035178

[b4] Wang Y. B. B., Lesburguère E., Leinekugel X., Liu W., Weinstein P. R. & Liu J. Environmental enrichment preserves cortical inputs to the parahippocampal areas and reduces post stroke diaschisis. Am J Neuroprotection & Neuroregeneration 3, 66–76, doi: 10.1166/ajnn.2011.1027 (2011).

[b5] Vann S. D., Brown M. W., Erichsen J. T. & Aggleton J. P. Fos imaging reveals differential patterns of hippocampal and parahippocampal subfield activation in rats in response to different spatial memory tests. The Journal of neuroscience: the official journal of the Society for Neuroscience 20, 2711–2718 (2000). 10729352 10.1523/JNEUROSCI.20-07-02711.2000 PMC6772240

[b6] Vann S. D., Brown M. W., Erichsen J. T. & Aggleton J. P. Using fos imaging in the rat to reveal the anatomical extent of the disruptive effects of fornix lesions. The Journal of neuroscience: the official journal of the Society for Neuroscience 20, 8144–8152 (2000). 11050137 10.1523/JNEUROSCI.20-21-08144.2000 PMC6772746

[b7] Jenkins T. A., Dias R., Amin E. & Aggleton J. P. Changes in Fos expression in the rat brain after unilateral lesions of the anterior thalamic nuclei. The European journal of neuroscience 16, 1425–1432 (2002). 12405955 10.1046/j.1460-9568.2002.02211.x

[b8] Holschneider D. P., Guo Y., Wang Z., Roch M. & Scremin O. U. Remote brain network changes after unilateral cortical impact injury and their modulation by acetylcholinesterase inhibition. Journal of neurotrauma 30, 907–919, doi: 10.1089/neu.2012.2657 (2013). 23343118 PMC3684212

[b9] Wang Z. . Functional reorganization of motor and limbic circuits after exercise training in a rat model of bilateral parkinsonism. PloS one 8, e80058, doi: 10.1371/journal.pone.0080058 (2013). 24278239 PMC3836982

[b10] Frankland P. W. & Bontempi B. The organization of recent and remote memories. Nature reviews. Neuroscience 6, 119–130, doi: 10.1038/nrn1607 (2005). 15685217

[b11] Schmitt O. & Eipert P. neuroVIISAS: approaching multiscale simulation of the rat connectome. Neuroinformatics 10, 243–267, doi: 10.1007/s12021-012-9141-6 (2012). 22350719

[b12] Schmitt O. . The intrinsic connectome of the rat amygdala. Frontiers in neural circuits 6, 81, doi: 10.3389/fncir.2012.00081 (2012). 23248583 PMC3518970

[b13] Sun H. . AAV-mediated netrin-1 overexpression increases peri-infarct blood vessel density and improves motor function recovery after experimental stroke. Neurobiology of disease 44, 73–83, doi: 10.1016/j.nbd.2011.06.006 (2011). 21726647 PMC3179859

[b14] Liu J., Nickolenko J. & Sharp F. R. Morphine induces c-fos and junB in striatum and nucleus accumbens via D1 and N-methyl-D-aspartate receptors. Proceedings of the National Academy of Sciences of the United States of America 91, 8537–8541 (1994). 8078918 10.1073/pnas.91.18.8537 PMC44641

[b15] Burns G. Neural connectivity of the rat: Theory, methods and applications Ph.D. thesis, University of Oxford (1997).

[b16] Sporns O. Networks of the brain (The MIT Press, 2011).

[b17] Stephan K. E. . Advanced database methodology for the Collation of Connectivity data on the Macaque brain (CoCoMac). Philos Trans R Soc Lond B Biol Sci 356, 1159–1186, doi: 10.1098/rstb.2001.0908 (2001). 11545697 PMC1088509

[b18] Paxinos G. W. C., The rat brain in stereotaxic coordinates. 6th edn (Academic Press, 2007).

[b19] Gould P. R. On the geographical interpretation of eigenvalues. Transactions of the Institute of British Geographers 42, 53–86 (1967).

[b20] Estrada E. Virtual identification of essential proteins within the protein interaction network of yeast. Proteomics 6, 35–40, doi: 10.1002/pmic.200500209 (2006). 16281187

[b21] Kleinberg J. M. Authoritative sources in a hyperlinked environment. Journal of the ACM 46, 604–632 (1999).

[b22] Schmitt O., Eipert P., Kettlitz R., Leßmann F. & Wree A. The connectome of the basal ganglia. Brain Struct Funct 221, 753–814 (2016). 25432770 10.1007/s00429-014-0936-0

[b23] Sun C. . Conditional ablation of neuroprogenitor cells in adult mice impedes recovery of poststroke cognitive function and reduces synaptic connectivity in the perforant pathway. The Journal of neuroscience: the official journal of the Society for Neuroscience 33, 17314–17325, doi: 10.1523/JNEUROSCI.2129-13.2013 (2013). 24174664 PMC3812503

[b24] Crofts J. J. . Network analysis detects changes in the contralesional hemisphere following stroke. NeuroImage 54, 161–169, doi: 10.1016/j.neuroimage.2010.08.032 (2011). 20728543 PMC3677803

[b25] Grefkes C. & Fink G. R. Reorganization of cerebral networks after stroke: new insights from neuroimaging with connectivity approaches. Brain: a journal of neurology 134, 1264–1276, doi: 10.1093/brain/awr033 (2011). 21414995 PMC3097886

[b26] Kataoka K. . Neuronal network disturbance after focal ischemia in rats. Stroke; a journal of cerebral circulation 20, 1226–1235 (1989). 10.1161/01.str.20.9.1226 2475923

[b27] Dijkhuizen R. M. . Correlation between brain reorganization, ischemic damage, and neurologic status after transient focal cerebral ischemia in rats: a functional magnetic resonance imaging study. The Journal of neuroscience: the official journal of the Society for Neuroscience 23, 510–517 (2003). 12533611 10.1523/JNEUROSCI.23-02-00510.2003 PMC6741861

[b28] Sagar S. M., Sharp F. R. & Curran T. Expression of c-fos protein in brain: metabolic mapping at the cellular level. Science 240, 1328–1331 (1988). 3131879 10.1126/science.3131879

[b29] Dragunow M. & Faull R. The use of c-fos as a metabolic marker in neuronal pathway tracing. J Neurosci Methods 29, 261–265 (1989). 2507830 10.1016/0165-0270(89)90150-7

[b30] Herrera D. G. & Robertson H. A. Activation of c-fos in the brain. Prog Neurobiol 50, 83–107 (1996). 8971979 10.1016/s0301-0082(96)00021-4

[b31] Herdegen T. & Leah J. D. Inducible and constitutive transcription factors in the mammalian nervous system: control of gene expression by Jun, Fos and Krox, and CREB/ATF proteins. Brain Res Brain Res Rev 28, 370–490 (1998). 9858769 10.1016/s0165-0173(98)00018-6

[b32] Tischmeyer W. & Grimm R. Activation of immediate early genes and memory formation. Cell Mol Life Sci 55, 564–574 (1999). 10357227 10.1007/s000180050315 PMC11146814

[b33] Vann S. D., Brown M. W. & Aggleton J. P. Fos expression in the rostral thalamic nuclei and associated cortical regions in response to different spatial memory tests. Neuroscience 101, 983–991 (2000). 11113347 10.1016/s0306-4522(00)00288-8

[b34] Friedman H. R. & Goldman-Rakic P. S. Activation of the hippocampus and dentate gyrus by working-memory: a 2-deoxyglucose study of behaving rhesus monkeys. J Neurosci 8, 4693–4706 (1988). 3199202 10.1523/JNEUROSCI.08-12-04693.1988 PMC6569568

[b35] Kilduff T. S., Miller J. D., Radeke C. M., Sharp F. R. & Heller H. C. 14C-2-deoxyglucose uptake in the ground squirrel brain during entrance to and arousal from hibernation. J Neuroscie 10, 2463–2475 (1990). 10.1523/JNEUROSCI.10-07-02463.1990 PMC6570375 2376782

[b36] Jones M. W. . A requirement for the immediate early gene Zif268 in the expression of late LTP and long-term memories. Nat Neurosci 4, 289–296 (2001). 11224546 10.1038/85138

[b37] Fleischmann A. . Impaired long-term memory and NR2A-type NMDA receptor-dependent synaptic plasticity in mice lacking c-Fos in the CNS. J Neurosci 23, 9116–9122 (2003). 14534245 10.1523/JNEUROSCI.23-27-09116.2003 PMC6740829

[b38] Lee J. L., Everitt B. J. & Thomas K. L. Independent cellular processes for hippocampal memory consolidation and reconsolidation. Science 304, 839–843 (2004). 15073322 10.1126/science.1095760

[b39] Grimm R. . Suppression of c-fos induction in rat brain impairs retention of a brightness discrimination reaction. Learn Mem 3, 402–413 (1997). 10456107 10.1101/lm.3.5.402

[b40] Maviel T., Durkin T. P., Menzaghi F. & Bontempi B. Sites of neocortical reorganization critical for remote spatial memory. Science 305, 96–99 (2004). 15232109 10.1126/science.1098180

[b41] Frankland P. W., Bontempi B., Talton L. E., Kaczmarek L. & Silva A. J. The involvement of the anterior cingulate cortex in remote contextual fear memory. Science 304, 881–883 (2004). 15131309 10.1126/science.1094804

[b42] Jenkins T. A., Dias R., Amin E., Brown M. W. & Aggleton J. P. Fos imaging reveals that lesions of the anterior thalamic nuclei produce widespread limbic hypoactivity in rats. The Journal of neuroscience: the official journal of the Society for Neuroscience 22, 5230–5238 (2002). 12077218 10.1523/JNEUROSCI.22-12-05230.2002 PMC6757752

[b43] Jenkins T. A., Vann S. D., Amin E. & Aggleton J. P. Anterior thalamic lesions stop immediate early gene activation in selective laminae of the retrosplenial cortex: evidence of covert pathology in rats? Eur J Neurosci 19, 3291–3304 (2004). 15217385 10.1111/j.0953-816X.2004.03421.x

[b44] Jenkins T. A., Amin E., Brown M. W. & Aggleton J. P. Changes in immediate early gene expression in the rat brain after unilateral lesions of the hippocampus. Neuroscience 137, 747–759, doi: 10.1016/j.neuroscience.2005.09.034 (2006). 16298079

[b45] Jenkins T. A., Amin E., Harold G. T., Pearce J. M. & Aggleton J. P. Distinct patterns of hippocampal formation activity associated with different spatial tasks: a Fos imaging study in rats. Experimental brain research 151, 514–523, doi: 10.1007/s00221-003-1499-0 (2003). 12830344

[b46] Jenkins T. A., Amin E., Pearce J. M., Brown M. W. & Aggleton J. P. Novel spatial arrangements of familiar visual stimuli promote activity in the rat hippocampal formation but not the parahippocampal cortices: a c-fos expression study. Neuroscience 124, 43–52, doi: 10.1016/j.neuroscience.2003.11.024 (2004). 14960338

[b47] Aggleton J. P. & Saunders R. C. The relationships between temporal lobe and diencephalic structures implicated in anterograde amnesia. Memory 5, 49–71 (1997). 9156091 10.1080/741941143

[b48] Parker A., Eacott M. J. & Gaffan D. The recognition memory deficit caused by mediodorsal thalamic lesion in non-human primates: a comparison with rhinal cortex lesion. Eur J Neurosci 9, 2423–2431 (1997). 9464936 10.1111/j.1460-9568.1997.tb01659.x

[b49] Aggleton J. P. & Brown M. W. Contrasting hippocampal and perirhinal cortex function using immediate early gene imaging. The Quarterly journal of experimental psychology. B, Comparative and physiological psychology 58, 218–233, doi: 10.1080/02724990444000131 (2005). 16194966

[b50] Balamotis M. A. . Satb1 ablation alters temporal expression of immediate early genes and reduces dendritic spine density during postnatal brain development. Molecular and cellular biology 32, 333–347, doi: 10.1128/MCB.05917-11 (2012). 22064485 PMC3255767

[b51] Huang Y. . Distribution of Satb1 in the central nervous system of adult mice. Neuroscience research 71, 12–21, doi: 10.1016/j.neures.2011.05.015 (2011). 21658419

[b52] Summers P. M. . Functional deficits induced by cortical microinfarcts. Journal of cerebral blood flow and metabolism: official journal of the International Society of Cerebral Blood Flow and Metabolism, 271678X16685573, doi: 10.1177/0271678X16685573 (2017). PMC5669342 28090802

[b53] Erdős P. & Rényi A. On random graphs. Publicationes Mathematicae 6, 290–297 (1959).

[b54] Okabe N. . Neural network remodeling underlying motor map reorganization induced by rehabilitative training after ischemic stroke. Neuroscience 339, 338–362 (2016). 27725217 10.1016/j.neuroscience.2016.10.008

[b55] Sugar J., Witter M. P., van Strien N. M. & Cappaert N. L. The retrosplenial cortex: intrinsic connectivity and connections with the (para)hippocampal region in the rat. An interactive connectome. Front Neuroinform 5, 7, doi: 10.3389/fninf.2011.00007 (2011). 21847380 PMC3147162

[b56] Kristensson K., Olsson Y. & Sjostrand J. Axonal uptake and retrograde transport of exogenous proteins in the hypoglossal nerve. Brain Res 32, 399–406 (1971). 4109164 10.1016/0006-8993(71)90332-5

