# Supplementary material for: Prediction of regional functional impairment following experimental stroke via connectome analysis
Source: Sci Rep. 2017 Apr 13;7:46316. doi: 10.1038/srep46316 (PMC5390322; doi:10.1038/srep46316)
Supplement: Supplementary Materials [file srep46316-s1.pdf]

Prediction of regional functional impairment following experimental stroke via connectome analysis

O. Schmitt[1], S. Badurek[2,3], W. Liu[2,3, 4], Y. Wang[2,3, 4], G.Rabiller[2,3], A. Kanoke [2,3], P. Eipert[1], J. Liu[2,3]

Department of Anatomy, University of Rostock[1], Germany.
Department of Neurological Surgery, UCSF[2] and SFVAMC[3], San Francisco, CA 94158
Department of Neurological Surgery, Beijing Tiantan Hospital[4], Capital Medical University, Beijing, PR China, 100050

Running title: Connectome analysis of stroke-induced changes in brain activation

Corresponding author: Dr. Jialing Liu, Department of Neurological Surgery (112C), University of California at San Francisco and Department of Veterans Affairs Medical Center, 1700 Owens Street, San Francisco, California 94158, USA.
Phone: (415) 575-0407; Fax: (415) 575-0595; E-mail: jialing.liu@ucsf.edu

Supplementary Materials

Supplementary Table 1. Abbreviations of Fos expressing regions used for connectome analysis. Frequently used alternative abbreviations of some regions are mentioned in parenthesis.

AcbSH/AcbC	Accumbens nucleus
ACCc	Anterior cingulate cortex caudal part (cACA)
ACCr	Anterior cingulate cortex rostral part (rACA)
AD	Anterodorsal thalamic nucleus
AGl	Lateral agranular prefrontal cortex (M1, MOP)
AID	Agranular insular cortex dorsal part
AIV	Agranular insular cortex ventral part
AM	Anteromedial thalamic nucleus
ArcM/ArcLP	Arcuate nucleus
Au1	Primary auditory cortex
AV	Anteroventral thalamic nucleus
BLA/CeM	Basolateral amygdaloid nucleus
CA1	Field CA1 of hippocampus
CA3	Field CA3 of hippocampus
CL/DEn	Claustrum
CPu	Caudate putamen
CxA	Amygdala cortex transition region
DG	Dentate gyrus
DI	Dysgranular insular cortex
GI	Granular insular cortex
IPAC	Interstitial nucleus of the posterior limb of the anterior commissure
LEnt	Lateral entorhinal cortex
LH/AHA	Lateral hypothalamus
LS	Lateral septum
MEnt	Medial entorhinal cortex
MG	Medial geniculate nucleus
MHb	Medial habenular nucleus
MPA/MPOL/MPOM	Medial preoptic area (MPA+MPOL+MPOM)
MPT	Medial pretectal nucleus
Op	Optic nerve layer of the superior colliculus
PaAP/PaPO	Paraventricular hypothalamic nucleus anterior parvicellular part (PaAP) and posterior part (PaPO)
PAG	Periaqueductal gray
Pir	Piriform cortex
PLSD	Protected least significant difference
PRh	Perirhinal cortex
PtA	Parietal association cortex
PV/PVA/PVP	Paraventricular thalamic nucleus
RCh	Retrochiasmatic area
RSGca	Retrosplenial granular cortex caudal part (=cRSP)
RSGr	Retrosplenial granular cortex rostral part (=rRSP)

S1	Primary somatosensory cortex
SCh	Suprachiasmatic nucleus
SO	Supraoptic nucleus
SuM	Supramammillar nucleus (SM)
VDB	Nucleus of the ventral limb of the diagonal band

Supplementary Table 2: Differences between home and exploration environment. It is assumed that the ipsilateral and contralateral Fos expressions of the sham-groups are equal.

	Home	Exploration	Home	Exploration
Ipsi	$\overline{Dr_H^{ipsi}} = 0.437$	$\overline{Dr_E^{ipsi}} = 0.288$	$\overline{D\sigma_H^{ipsi}} = 2.244$	$\overline{D\sigma_E^{ipsi}} = 2.427$
Contra	$\overline{Dr_E^{contra}} = 0.344$	$\overline{Dr_E^{contra}} = 0.175$	$\overline{D\sigma_H^{contra}} = 1.651$	$\overline{D\sigma_E^{contra}} = 1.334$

The environment has a remarkable effect on the Fos expression. The mean Fos expression of the regions of the sham-and dMCAO-animals in the exploration environment is on average about four (295%) and six (549% ipsilateral and 589% contralateral) times larger with regard to the animals in the home environment, respectively. D_r is the relative difference of Fos expression of the ipsilateral side (formal definition see Material and Methods).

Supplementary Table 3: Mean differences and standard deviations (σ) over all regions between the sham and dMCAO groups.

	Sham	dMCAO	Sham	dMCAO
ipsi	$\overline{Dr_s^{ipsi}} = 2.955$	$\overline{Dr_m^{ipsi}} = 5.488$	$\overline{D\sigma_s^{ipsi}} = 13.642$	$\overline{D\sigma_m^{ipsi}} = 19.258$
contra	$\overline{Dr_s^{contra}} = 2.955$	$\overline{Dr_m^{contra}} = 5.885$	$\overline{D\sigma_s^{contra}} = 13.642$	$\overline{D\sigma_m^{contra}} = 24.933$

The ipsilateral regions of the dMCAO-animals in the home as well as in the exploration condition show a larger mean change of the Fos expression (44% resp. 0.437) than the contralateral regions (34%).Only 7 (home) and 6 (exploration) of the 38 ipsilateral regions are hyper-activated, compared to 11 (home) and 19 (exploration) regions contralaterally.

Supplementary Table 4: Relative Fos differences of the home (left) and the exploration group (right). Increased Fos changes are shown as positive, while decreased as negative. The regions were sorted by their Fos changes (L: left hemisphere, R: right hemisphere).

Side	Region	Home sham vs. dMCAO	Side	Region	Exp sham vs. dMCAO
L	Medial pretectal nucleus	0.469	L	Nucleus of the vertical limb of the diagonal band	0.497
L	Lateral hypothalamic area and anterior hypothalamic area	0.178	L	Nucleus paraventricularis anterior and posterior part	0.256
L	Nucleus paraventricularis anterior and posterior part	0.174	L	Suprachiasmatic nucleus	0.078
L	Arcuate nucleus medial part and arcuate nucleus lateroposterior part	0.150	L	Accumbens core shell	0.076
L	Suprachiasmatic nucleus	0.141	L	Lateral hypothalamic area and anterior hypothalamic area	0.050
L	Retrochiasmatic area	0.141	L	Optic nerve layer of the superior colliculus	0.012
L	Perirhinal cortex	0.020	L	Arcuate nucleus medial part and arcuate nucleus lateroposterior part	-0.012
L	Supraoptic nucleus [Supraoptic region]	-0.100	L	Retrochiasmatic area	-0.048
L	Cortex amygdala transition zone	-0.137	L	Lateral septal nucleus	-0.079
L	Nucleus of the vertical limb of the diagonal band	-0.172	L	Supramammillar nucleus	-0.111
L	Paraventricular nucleus anterior posterior parts	-0.181	L	Field CA3 of hippocampus	-0.118
L	Medial habenular nucleus	-0.191	L	Paraventricular nucleus anterior posterior parts	-0.132
L	Optic nerve layer of the superior colliculus	-0.230	L	Caudate putamen	-0.145
L	Claustrum and dorsal endopirifom nucleus	-0.323	L	Medial habenular nucleus	-0.185
L	Basolateral and central medial amygdala	-0.350	L	Medial preoptic area and medial preoptic nucleus lateral and medial	-0.201
L	Lateral septal nucleus	-0.360	L	Interstitial nucleus of the posterior limb of the anterior commissure	-0.218
L	Accumbens core shell	-0.374	L	Medial pretectal nucleus	-0.225
L	Interstitial nucleus of the posterior limb of the anterior commissure	-0.374	L	Dentate gyrus	-0.257
L	Piriform cortex	-0.411	L	Anteroventral thalamic nucleus	-0.288
L	Supramammillar nucleus	-0.476	L	Basolateral and central medial amygdala	-0.291
L	Medial preoptic area and medial preoptic nucleus lateral and medial	-0.477	L	Medial entorhinal cortex	-0.291
L	Retrosplenial granular cortex caudal part	-0.492	L	Lateral agranular prefrontal cortex	-0.299
L	Field CA3 of hippocampus	-0.523	L	Supraoptic nucleus [Supraoptic region]	-0.303
L	Caudate putamen	-0.526	L	Retrosplenial granular cortex caudal part	-0.311
L	Lateral entorhinal cortex	-0.543	L	Field CA1 of hippocampus	-0.326
L	Medial entorhinal cortex	-0.547	L	Claustrum and dorsal endopirifom nucleus	-0.330
L	Dentate gyrus	-0.574	L	Primary auditory cortex	-0.358
L	Anterior cingulate cortex rostral part	-0.585	L	Anteromedial thalamic nucleus	-0.358
L	Primary auditory cortex	-0.620	L	Periaqueductal gray	-0.370
L	Field CA1 of hippocampus	-0.621	L	Retrosplenial granular cortex rostral part	-0.374
L	Anteroventral thalamic nucleus	-0.685	L	Medial geniculate nucleus	-0.379
L	Anteromedial thalamic nucleus	-0.687	L	Cortex amygdala transition zone	-0.468
L	Medial geniculate nucleus	-0.739	L	Anterior cingulate cortex rostral part	-0.473
L	Retrosplenial granular cortex rostral part	-0.745	L	Lateral entorhinal cortex	-0.486
L	Periaqueductal gray	-0.754	L	Perirhinal cortex	-0.541
L	Anterior cingulate cortex caudal part	-0.804	L	Anterior cingulate cortex caudal part	-0.606
L	Anterodorsal thalamic nucleus	-0.831	L	Anterodorsal thalamic nucleus	-0.653
L	Lateral agranular prefrontal cortex	-0.903	L	Piriform cortex	-0.737
R	Medial pretectal nucleus	0.484	R	Nucleus of the vertical limb of the diagonal band	0.543
R	Piriform cortex	0.373	R	Nucleus paraventricularis anterior and posterior part	0.390
R	Cortex amygdala transition zone	0.320	R	Primary auditory cortex	0.376
R	Primary auditory cortex	0.237	R	Suprachiasmatic nucleus	0.330
R	Nucleus of the vertical limb of the diagonal band	0.167	R	Retrosplenial granular cortex caudal part	0.220
R	Nucleus paraventricularis anterior and posterior part	0.149	R	Accumbens core shell	0.203
R	Accumbens core shell	0.114	R	Retrosplenial granular cortex rostral part	0.200
R	Lateral hypothalamic area and anterior hypothalamic area	0.060	R	Lateral agranular prefrontal cortex	0.194
R	Arcuate nucleus medial part and arcuate nucleus lateroposterior part	0.035	R	Lateral hypothalamic area and anterior hypothalamic area	0.193
R	Suprachiasmatic nucleus	0.016	R	Caudate putamen	0.187
R	Medial habenular nucleus	0.001	R	Medial entorhinal cortex	0.128
R	Basolateral and central medial amygdala	-0.003	R	Optic nerve layer of the superior colliculus	0.122
R	Paraventricular nucleus anterior posterior parts	-0.082	R	Arcuate nucleus medial part and arcuate nucleus lateroposterior part	0.070
R	Optic nerve layer of the superior colliculus	-0.084	R	Interstitial nucleus of the posterior limb of the anterior commissure	0.054
R	Supraoptic nucleus [Supraoptic region]	-0.161	R	Claustrum and dorsal endopirifom nucleus	0.053
R	Retrochiasmatic area	-0.167	R	Supramammillar nucleus	0.049
R	Perirhinal cortex	-0.180	R	Lateral septal nucleus	0.039
R	Claustrum and dorsal endopirifom nucleus	-0.206	R	Medial preoptic area and medial preoptic nucleus lateral and medial	0.026
R	Supramammillar nucleus	-0.218	R	Piriform cortex	0.000
R	Interstitial nucleus of the posterior limb of the anterior commissure	-0.232	R	Basolateral and central medial amygdala	-0.034
R	Lateral septal nucleus	-0.275	R	Anteroventral thalamic nucleus	-0.038
R	Retrosplenial granular cortex caudal part	-0.320	R	Field CA1 of hippocampus	-0.041
R	Field CA3 of hippocampus	-0.332	R	Field CA3 of hippocampus	-0.047
R	Retrosplenial granular cortex rostral part	-0.343	R	Anterior cingulate cortex rostral part	-0.068
R	Medial entorhinal cortex	-0.351	R	Dentate gyrus	-0.068
R	Caudate putamen	-0.370	R	Medial pretectal nucleus	-0.074
R	Medial preoptic area and medial preoptic nucleus lateral and medial	-0.421	R	Anterior cingulate cortex caudal part	-0.085
R	Dentate gyrus	-0.494	R	Paraventricular nucleus anterior posterior parts	-0.086
R	Lateral entorhinal cortex	-0.542	R	Medial habenular nucleus	-0.146
R	Anterior cingulate cortex rostral part	-0.558	R	Retrochiasmatic area	-0.149

R	Lateral agranular prefrontal cortex	-0.646	R	Cortex amygdala transition zone	-0.166
R	Field CA1 of hippocampus	-0.672	R	Anteromedial thalamic nucleus	-0.206
R	Anterior cingulate cortex caudal part	-0.692	R	Lateral entorhinal cortex	-0.216
R	Medial geniculate nucleus	-0.702	R	Perirhinal cortex	-0.220
R	Anteroventral thalamic nucleus	-0.722	R	Medial geniculate nucleus	-0.297
R	Anteromedial thalamic nucleus	-0.729	R	Periaqueductal gray	-0.301
R	Periaqueductal gray	-0.740	R	Supraoptic nucleus [Supraoptic region]	-0.480
R	Anterodorsal thalamic nucleus	-0.855	R	Anterodorsal thalamic nucleus	-0.534

Supplementary Table 5: Correlation coefficients for the correlation between the Fos changes and the spatial distances of dMCAO-damaged region to regions that show Fos changes.

	PtA	AID	AIV	DI	GI	S1
Home	0.447	0.085	0.059	0.140	0.156	0.328
Exploration	0.356	0.196	0.148	0.308	0.322	0.442
Home, Exploration	0.469	0.153	0.113	0.245	0.263	0.435
Ipsi home	0.498	0.094	0.065	0.139	0.159	0.346
Ipsi exploration	0.403	0.151	0.096	0.253	0.274	0.363
Ipsi home, exploration	0.525	0.135	0.089	0.215	0.238	0.405
Contra home	0.330	-0.087	-0.101	-0.057	-0.040	0.203
Contra exploration	0.035	-0.192	-0.211	-0.153	-0.154	0.061
Contra home, exploration	0.250	-0.150	-0.169	-0.111	-0.100	0.172

Supplementary Table 6: Local network parameters of regions with Fos changes. The Fos changes are standing in the Home column for Fos changes of home cage animals and dMCAO, The Fos changes are of the Exploration cage animals versus dMCAO are in the Expl. Column. AvgDg is the average number inputs and outputs of the network including the regions that were damaged following dMCAO. The damaged column to the right of AvgDg contains the AvgDg values of the network where dMCAO damaged regions were removed. SubCen: normalized subgraph centrality.

Side	Region	AvgDG	damaged	SubCen	damaged	Hubness	damaged	Authority	damaged	Home	Expl.
L	Accumbens core shell	233.74	232.66	0.0721	0.0330	0.1441	0.1432	0.4024	0.3980	-0.374	0.076
L	Anteromedial thalamic nucleus	200.09	198.41	0.0541	0.0242	0.1512	0.1471	0.2968	0.2946	-0.687	-0.358
L	Anteroventral thalamic nucleus	202.92	201.18	0.0656	0.0293	0.1553	0.1519	0.3344	0.3288	-0.685	-0.288
L	Basolateral and central medial amygdala	167.30	165.65	0.4752	0.2109	0.5913	0.5779	0.6585	0.6496	-0.350	-0.291
L	Caudate putamen	170.91	169.04	0.1871	0.0826	0.2287	0.2240	0.6839	0.6667	-0.526	-0.145
L	Claustrum and dorsal endopirifom nucleus	211.14	208.33	0.1163	0.0506	0.3113	0.3037	0.2772	0.2657	-0.323	-0.330
L	Interstitial nucleus of the posterior limb of the anterior commissure	222.69	220.83	0.0797	0.0355	0.2034	0.1995	0.3297	0.3243	-0.374	-0.218
L	Lateral agranular prefrontal cortex	173.70	171.13	0.1959	0.0863	0.4509	0.4429	0.3374	0.3229	-0.903	-0.299
L	Lateral entorhinal cortex	185.04	183.75	0.1062	0.0435	0.2414	0.2282	0.3731	0.3570	-0.543	-0.486
L	Lateral hypothalamic area and anterior hypothalamic area	144.69	143.20	1.0000	0.4513	0.9713	0.9624	0.9013	0.8882	0.178	0.050
L	Medial entorhinal cortex	174.05	172.45	0.0489	0.0190	0.1423	0.1318	0.3160	0.3004	-0.547	-0.291
L	Medial geniculate nucleus	203.58	202.08	0.0546	0.0226	0.2007	0.1910	0.2241	0.2142	-0.739	-0.379
L	Medial preoptic area and medial preoptic nucleus lateral and medial	194.31	192.80	0.3015	0.1392	0.5206	0.5217	0.5258	0.5216	-0.477	-0.201
L	Paraventricular nucleus anterior posterior parts	199.61	198.49	0.3472	0.1579	0.4912	0.4858	0.5846	0.5812	-0.181	-0.132
L	Periaqueductal gray	157.59	156.19	0.8551	0.3904	0.8796	0.8769	0.9126	0.9012	-0.754	-0.370
L	Perirhinal cortex	178.64	176.84	0.1739	0.0732	0.3903	0.3760	0.3589	0.3394	0.020	-0.541
L	Piriform cortex	201.61	200.42	0.1262	0.0561	0.2720	0.2669	0.4015	0.3937	-0.411	-0.737
L	Primary auditory cortex	210.15	204.94	0.0099	0.0043	0.1197	0.1178	0.0685	0.0629	-0.620	-0.358
L	Retrosplenial granular cortex caudal part	178.21	174.50	0.0087	0.0038	0.0882	0.0875	0.0767	0.0722	-0.492	-0.311
L	Supramammillar nucleus	197.86	195.58	0.2043	0.0933	0.4665	0.4630	0.4183	0.4167	-0.476	-0.111
L	Anterior cingulate cortex caudal part	207.00	203.17	0.0001	0.0001	0.0101	0.0100	0.0054	0.0052	-0.804	-0.606
L	Anterior cingulate cortex rostral part	196.78	193.56	0.0001	0.0001	0.0213	0.0209	0.0045	0.0045	-0.585	-0.473
L	Anterodorsal thalamic nucleus	202.00	200.09	0.0155	0.0071	0.0506	0.0500	0.2421	0.2437	-0.831	-0.653
L	Arcuate nucleus medial part and arcuate nucleus lateroposterior part	256.61	255.89	0.0005	0.0002	0.0647	0.0652	0.0115	0.0115	0.150	-0.012
L	Cortex amygdala transition zone	276.75	274.07	0.0020	0.0009	0.1155	0.1156	0.0133	0.0133	-0.137	-0.468
L	Dentate gyrus	190.64	188.63	0.0456	0.0211	0.1428	0.1429	0.2554	0.2566	-0.574	-0.257
L	Field CA1 of hippocampus	170.07	168.11	0.1117	0.0505	0.2973	0.2916	0.2997	0.3005	-0.621	-0.326

L	Field CA3 of hippocampus	196.96	194.87	0.0231	0.0107	0.0806	0.0805	0.2571	0.2581	-0.523	-0.118
L	Lateral septal nucleus	166.59	164.91	0.3115	0.1446	0.4658	0.4656	0.5803	0.5833	-0.360	-0.079
L	Medial habenular nucleus	263.41	261.29	0.0327	0.0153	0.1669	0.1671	0.1707	0.1717	-0.191	-0.185
L	Medial pretectal nucleus	232.57	230.17	0.0043	0.0020	0.0812	0.0811	0.0605	0.0608	0.469	-0.225
L	Nucleus of the vertical limb of the diagonal band	213.59	211.06	0.1317	0.0599	0.3189	0.3150	0.3480	0.3489	-0.172	0.497
L	Nucleus paraventricularis anterior and posterior part	258.53	256.33	0.0099	0.0046	0.0578	0.0579	0.1961	0.1977	0.174	0.256
L	Optic nerve layer of the superior colliculus	155.94	153.87	0.0019	0.0009	0.0466	0.0464	0.0388	0.0387	-0.230	0.012
L	Retrochiasmatic area	216.40	214.66	0.0878	0.0409	0.2402	0.2397	0.3428	0.3455	0.141	-0.048
L	Retrosplenial granular cortex rostral part	258.13	253.46	0.0040	0.0018	0.0813	0.0803	0.0319	0.0316	-0.745	-0.374
L	Suprachiasmatic nucleus	195.38	193.64	0.1155	0.0539	0.2766	0.2771	0.3860	0.3885	0.141	0.078
L	Supraoptic nucleus [Supraoptic region]	213.77	212.24	0.0567	0.0265	0.0988	0.0992	0.4651	0.4688	-0.100	-0.303
R	Accumbens core shell	233.74	233.09	0.0721	0.0340	0.1441	0.1454	0.4024	0.4034	0.114	0.203
R	Basolateral and central medial amygdala	167.30	166.28	0.4752	0.2192	0.5913	0.5877	0.6585	0.6594	-0.003	-0.034
R	Caudate putamen	170.91	169.52	0.1871	0.0861	0.2287	0.2302	0.6839	0.6723	-0.370	0.187
R	Lateral agranular prefrontal cortex	173.70	171.56	0.1959	0.0893	0.4509	0.4455	0.3374	0.3351	-0.646	0.194
R	Lateral hypothalamic area and anterior hypothalamic area	144.69	143.69	1.0000	0.4684	0.9713	0.9733	0.9013	0.9085	0.060	0.193
R	Periaqueductal gray	157.59	156.57	0.8551	0.3996	0.8796	0.8824	0.9126	0.9161	-0.740	-0.301
R	Perirhinal cortex	178.64	177.18	0.1739	0.0785	0.3903	0.3844	0.3589	0.3561	-0.180	-0.220
R	Anterior cingulate cortex caudal part	207.00	205.17	0.0001	0.0001	0.0101	0.0101	0.0054	0.0054	-0.692	-0.085
R	Anterior cingulate cortex rostral part	196.78	194.33	0.0001	0.0001	0.0213	0.0210	0.0045	0.0046	-0.558	-0.068
R	Anterodorsal thalamic nucleus	202.00	201.09	0.0155	0.0073	0.0506	0.0510	0.2421	0.2450	-0.855	-0.534
R	Anteromedial thalamic nucleus	200.09	198.92	0.0541	0.0254	0.1512	0.1516	0.2968	0.2997	-0.729	-0.206
R	Anteroventral thalamic nucleus	202.92	201.80	0.0656	0.0309	0.1553	0.1562	0.3344	0.3381	-0.722	-0.038
R	Arcuate nucleus medial part and arcuate nucleus lateroposterior part	256.61	255.67	0.0005	0.0003	0.0647	0.0655	0.0115	0.0116	0.035	0.070
R	Claustrum and dorsal endopirifom nucleus	211.14	209.52	0.1163	0.0535	0.3113	0.3077	0.2772	0.2800	-0.206	0.053
R	Cortex amygdala transition zone	276.75	275.36	0.0020	0.0010	0.1155	0.1163	0.0133	0.0136	0.320	-0.166
R	Dentate gyrus	190.64	189.63	0.0456	0.0215	0.1428	0.1440	0.2554	0.2581	-0.494	-0.068
R	Field CA1 of hippocampus	170.07	168.91	0.1117	0.0526	0.2973	0.2989	0.2997	0.3029	-0.672	-0.041
R	Field CA3 of hippocampus	196.96	195.95	0.0231	0.0109	0.0806	0.0812	0.2571	0.2598	-0.332	-0.047
R	Interstitial nucleus of the posterior limb of the anterior commissure	222.69	221.09	0.0797	0.0377	0.2034	0.2047	0.3297	0.3330	-0.232	0.054
R	Lateral entorhinal cortex	185.04	183.86	0.1062	0.0501	0.2414	0.2424	0.3731	0.3772	-0.542	-0.216
R	Lateral septal nucleus	166.59	165.66	0.3115	0.1470	0.4658	0.4689	0.5803	0.5874	-0.275	0.039
R	Medial entorhinal cortex	174.05	172.95	0.0489	0.0231	0.1423	0.1429	0.3160	0.3191	-0.351	0.128
R	Medial geniculate nucleus	203.58	201.77	0.0546	0.0251	0.2007	0.1993	0.2241	0.2265	-0.702	-0.297
R	Medial habenular nucleus	263.41	262.21	0.0327	0.0155	0.1669	0.1682	0.1707	0.1729	0.001	-0.146
R	Medial preoptic area and medial preoptic nucleus lateral and medial	194.31	193.38	0.3015	0.1424	0.5206	0.5241	0.5258	0.5326	-0.421	0.026
R	Medial pretectal nucleus	232.57	231.67	0.0043	0.0021	0.0812	0.0819	0.0605	0.0614	0.484	-0.074
R	Nucleus of the vertical limb of the diagonal band	213.59	212.58	0.1317	0.0623	0.3189	0.3213	0.3480	0.3528	0.167	0.543
R	Nucleus paraventricularis anterior and posterior part	258.53	257.15	0.0099	0.0047	0.0578	0.0580	0.1961	0.1985	0.149	0.390
R	Optic nerve layer of the superior colliculus	155.94	154.52	0.0019	0.0009	0.0466	0.0467	0.0388	0.0391	-0.084	0.122
R	Paraventricular nucleus anterior posterior parts	199.61	198.32	0.3472	0.1636	0.4912	0.4932	0.5846	0.5921	-0.082	-0.086
R	Piriform cortex	201.61	199.68	0.1262	0.0584	0.2720	0.2702	0.4015	0.4054	0.373	0.000
R	Primary auditory cortex	210.15	208.28	0.0099	0.0047	0.1197	0.1196	0.0685	0.0690	0.237	0.376
R	Retrochiasmatic area	216.40	215.32	0.0878	0.0415	0.2402	0.2419	0.3428	0.3470	-0.167	-0.149
R	Retrosplenial granular cortex caudal part	178.21	176.28	0.0087	0.0041	0.0882	0.0880	0.0767	0.0772	-0.320	0.220
R	Retrosplenial granular cortex rostral part	258.13	256.13	0.0040	0.0019	0.0813	0.0813	0.0319	0.0322	-0.343	0.200
R	Suprachiasmatic nucleus	195.38	194.23	0.1155	0.0544	0.2766	0.2784	0.3860	0.3906	0.016	0.330
R	Supramammillar nucleus	197.86	196.75	0.2043	0.0964	0.4665	0.4697	0.4183	0.4235	-0.218	0.049
R	Supraoptic nucleus [Supraoptic region]	213.77	212.71	0.0567	0.0267	0.0988	0.0994	0.4651	0.4706	-0.161	-0.480

Supplementary Table 7: Vulnerability of regions of the bilateral network of regions with Fos changes and the left hemispheric damaged regions. The regions are sorted by descending order of vulnerability. If the vulnerability value is very large or towards maximal as in the case of the Lateral hypothalamic area and anterior hypothalamic area (1.8), then a removal or damage of this region would enlarge the mean graph theoretical distance among the resting regions.

.

Side	Region	Vulnerability
L	Lateral hypothalamic area and anterior hypothalamic area	1.8301
R	Lateral hypothalamic area and anterior hypothalamic area	1.5741
L	Anteromedial thalamic nucleus	1.0696
R	Anteromedial thalamic nucleus	1.0202
L	Nucleus of the vertical limb of the diagonal band	0.9836
R	Nucleus of the vertical limb of the diagonal band	0.9648
L	Medial geniculate nucleus	0.7816
L	Periaqueductal gray	0.7656
L	Basolateral and central medial amygdala	0.7219
R	Periaqueductal gray	0.7069
R	Basolateral and central medial amygdala	0.6914
R	Medial geniculate nucleus	0.6735
L	Lateral septal nucleus	0.6444
R	Lateral septal nucleus	0.6350
L	Accumbens core shell	0.6200
L	Lateral agranular prefrontal cortex	0.6026

R	Accumbens core shell	0.5847
R	Lateral agranular prefrontal cortex	0.5697
L	Lateral entorhinal cortex	0.5575
L	Caudate putamen	0.5105
R	Caudate putamen	0.5082
L	Perirhinal cortex	0.4941
R	Perirhinal cortex	0.4424
L	Field CA1 of hippocampus	0.4377
L	Medial entorhinal cortex	0.4236
R	Field CA1 of hippocampus	0.3954
L	Paraventricular nucleus anterior posterior parts	0.3860
R	Lateral entorhinal cortex	0.3743
L	Supramammillar nucleus	0.3625
R	Supramammillar nucleus	0.3296
R	Paraventricular nucleus anterior posterior parts	0.3155
L	Medial preoptic area and medial preoptic nucleus lateral and medial	0.2803
R	Medial entorhinal cortex	0.2686
L	Primary somatosensory cortex	0.2686
R	Medial preoptic area and medial preoptic nucleus lateral and medial	0.2404
L	Piriform cortex	0.2145
L	Suprachiasmatic nucleus	0.2028
L	Parietal association cortex	0.1934
R	Suprachiasmatic nucleus	0.1934
R	Piriform cortex	0.1629
L	Anteroventral thalamic nucleus	0.1394
L	Claustrum and dorsal endopirifom nucleus	0.0854
R	Anteroventral thalamic nucleus	0.0689
L	Dysgranular insular cortex	0.0501
L	Dentate gyrus	0.0478
R	Claustrum and dorsal endopirifom nucleus	0.0384
R	Dentate gyrus	0.0337
R	Field CA3 of hippocampus	0.0337
L	Field CA3 of hippocampus	0.0337
R	Supraoptic nucleus [Supraoptic region]	0.0290
L	Supraoptic nucleus [Supraoptic region]	0.0290
L	Retrochiasmatic area	0.0125
R	Retrochiasmatic area	0.0055
L	Interstitial nucleus of the posterior limb of the anterior commissure	-0.0203
L	Anterodorsal thalamic nucleus	-0.0227
R	Anterodorsal thalamic nucleus	-0.0391
L	Agranular insular cortex dorsal part	-0.0556
L	Granular insular cortex	-0.0814
R	Interstitial nucleus of the posterior limb of the anterior commissure	-0.0838
L	Primary auditory cortex	-0.1636
R	Primary auditory cortex	-0.1918
L	Agranular insular cortex ventral part	-0.2130
L	Retrosplenial granular cortex caudal part	-0.2177
R	Medial habenular nucleus	-0.2811
L	Medial habenular nucleus	-0.2811
R	Retrosplenial granular cortex caudal part	-0.2858
L	Retrosplenial granular cortex rostral part	-0.3422
R	Retrosplenial granular cortex rostral part	-0.3656
L	Medial pretectal nucleus	-0.4079
R	Medial pretectal nucleus	-0.4455
L	Nucleus paraventricularis anterior and posterior part	-0.5944
R	Nucleus paraventricularis anterior and posterior part	-0.6156
L	Anterior cingulate cortex rostral part	-0.6207
R	Anterior cingulate cortex rostral part	-0.6254
L	Optic nerve layer of the superior colliculus	-0.6560
R	Optic nerve layer of the superior colliculus	-0.6771
L	Cortex amygdala transition zone	-0.6912
R	Cortex amygdala transition zone	-0.7194
L	Anterior cingulate cortex caudal part	-0.7523
R	Anterior cingulate cortex caudal part	-0.7593
L	Arcuate nucleus medial part and arcuate nucleus lateroposterior part	-1.5166
R	Arcuate nucleus medial part and arcuate nucleus lateroposterior part	-1.5190

Supplementary Figures

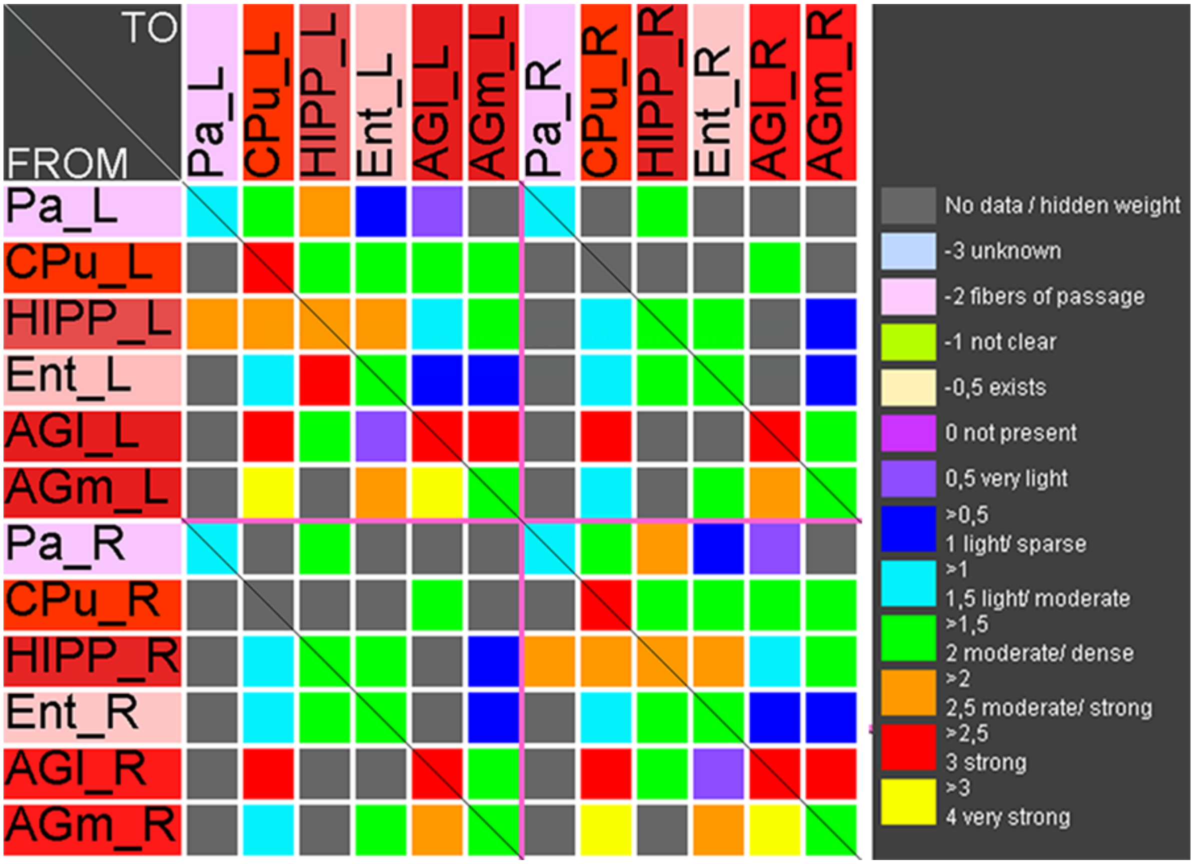

Supplementary Figure 1. Caudate putamen receives many inputs from the contralateral hemisphere. The bihemispheric adjacency matrix shows directional, weighted connections within and among the selected regions, subdivided by ipsi (L) and contralesional (R) quadrants (rose cross; major and minor diagonals in black). The overall density of connection within one hemisphere is greater than that across the hemisphere, yet the cross-hemispheric connections are relatively dense among the caudate-putamen and motor cortex. In particular, the caudate-putamen receives many contralateral inputs. The degree of known connections is coded by colour and the strength listed in an ascending order.

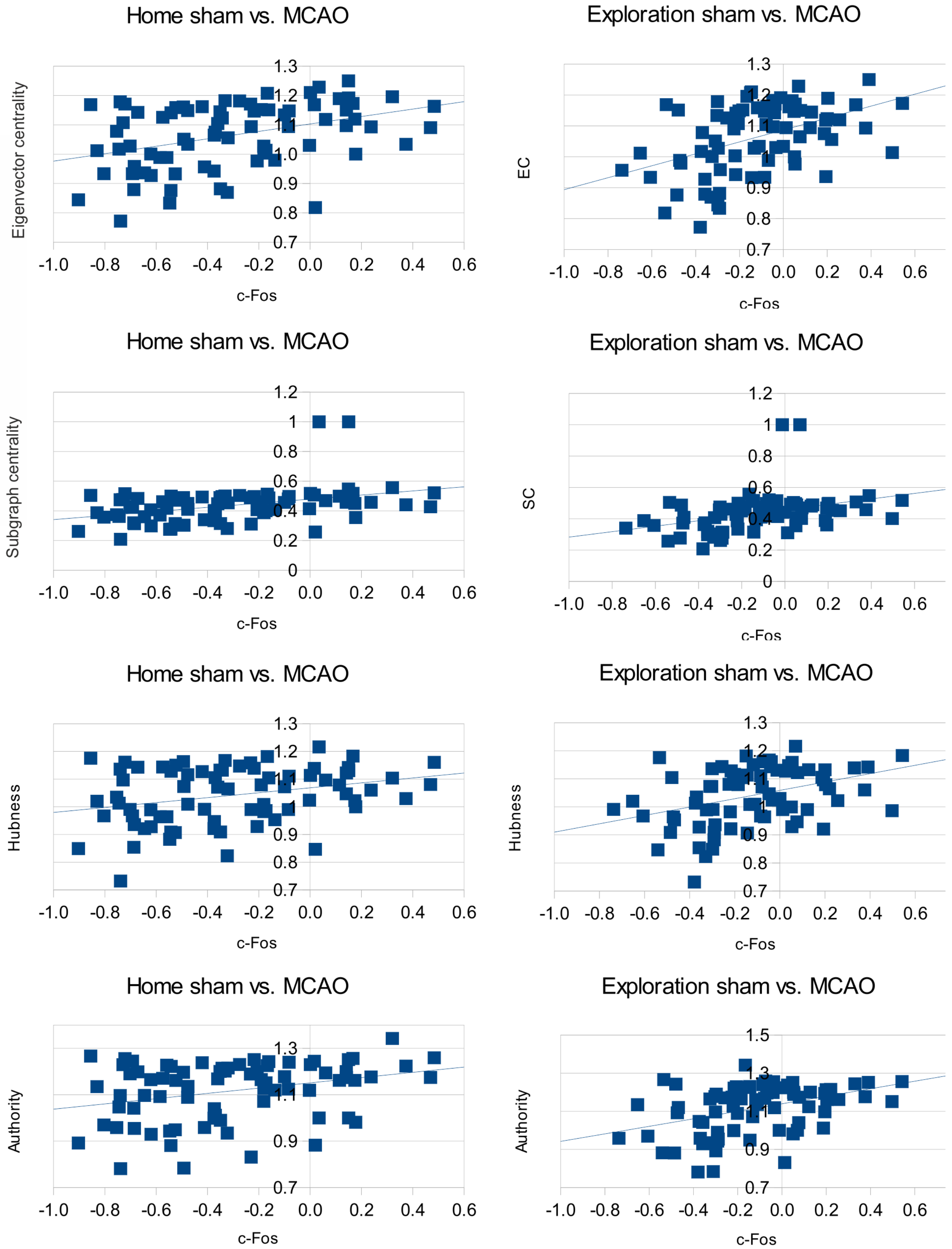
Home sham vs. MCAO
Eigenvector centrality
c-Fos
Exploration sham vs. MCAO
EC
c-Fos
Home sham vs. MCAO
Subgraph centrality
c-Fos
Exploration sham vs. MCAO
SC
c-Fos
Home sham vs. MCAO
Hubness
c-Fos
Exploration sham vs. MCAO
Hubness
c-Fos
Home sham vs. MCAO
Authority
c-Fos
Exploration sham vs. MCAO
Authority
c-Fos

Supplementary Figure 2. Correlation analyses of the local network parameters and Fos expression. Correlations of local parameters (ordinate) and Fos expression (abscissa) in a bilateral partial connectome that consists of only regions with Fos changes in home cage and exploration groups between sham and MCAO animals. Four parameters with the largest correlation coefficient are shown, as eigenvector centrality (EC), subgraph centrality (SC), Hubness and Authoritativeness (Authority).

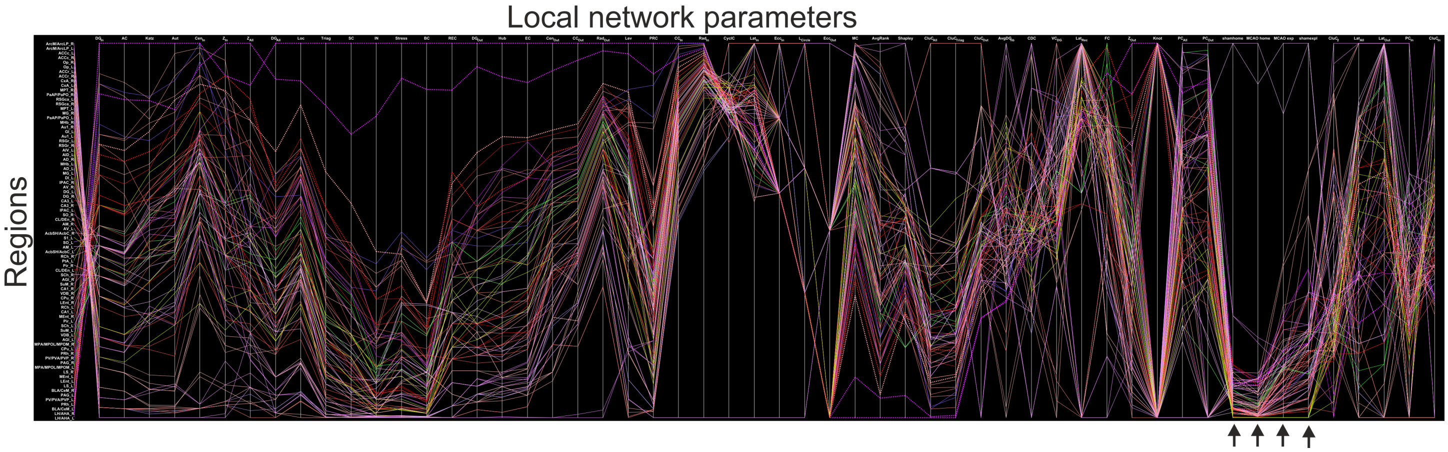

Supplementary Figure 3. Many regions in the Fos network exhibit similarity in local parameters. The similarity of local parameters (X-axis) over Fos-expressing regions (Y-axis) is presented in the parallel coordinate representation in combination with the mean Fos intensities of the 4 experimental groups of animals (arrows). The dashed lines mark the regions with highest 4 ranks. Rank 1: lateral hypothalamic area left hemisphere, rank 2: lateral hypothalamic area right hemisphere, rank 3: basolateral and central medial amygdala, rank 4: perirhinal cortex. Solid lines mark the remaining regions.
